# Tutorial: using NEURON for neuromechanical simulations

**DOI:** 10.3389/fncom.2023.1143323

**Published:** 2023-07-31

**Authors:** Chris Fietkiewicz, Robert A. McDougal, David Corrales Marco, Hillel J. Chiel, Peter J. Thomas

**Affiliations:** ^1^Department of Mathematics and Computer Science, Hobart and William Smith Colleges, Geneva, NY, United States; ^2^Department of Biostatistics, Yale School of Public Health, New Haven, CT, United States; ^3^Wu Tsai Institute, Yale University, New Haven, CT, United States; ^4^Program in Computational Biology and Bioinformatics, Yale University, New Haven, CT, United States; ^5^Section for Biomedical Informatics, Yale School of Medicine, New Haven, CT, United States; ^6^Department of Biology, Case Western Reserve University, Cleveland, OH, United States; ^7^Department of Neurosciences, Case Western Reserve University, Cleveland, OH, United States; ^8^Department of Biomedical Engineering, Case Western Reserve University, Cleveland, OH, United States; ^9^Department of Mathematics, Applied Mathematics and Statistics, Case Western Reserve University, Cleveland, OH, United States; ^10^Department of Cognitive Science, Case Western Reserve University, Cleveland, OH, United States; ^11^Department of Electrical, Control, and Systems Engineering, Case Western Reserve University, Cleveland, OH, United States; ^12^Department of Data and Computer Science, Case Western Reserve University, Cleveland, OH, United States

**Keywords:** brain, body, motor control, neural network, closed-loop, biomechanics

## Abstract

The dynamical properties of the brain and the dynamics of the body strongly influence one another. Their interaction generates complex adaptive behavior. While a wide variety of simulation tools exist for neural dynamics or biomechanics separately, there are few options for integrated brain-body modeling. Here, we provide a tutorial to demonstrate how the widely-used NEURON simulation platform can support integrated neuromechanical modeling. As a first step toward incorporating biomechanics into a NEURON simulation, we provide a framework for integrating inputs from a “periphery” and outputs to that periphery. In other words, “body” dynamics are driven in part by “brain” variables, such as voltages or firing rates, and “brain” dynamics are influenced by “body” variables through sensory feedback. To couple the “brain” and “body” components, we use NEURON's *pointer* construct to share information between “brain” and “body” modules. This approach allows separate specification of brain and body dynamics and code reuse. Though simple in concept, the use of pointers can be challenging due to a complicated syntax and several different programming options. In this paper, we present five different computational models, with increasing levels of complexity, to demonstrate the concepts of code modularity using pointers and the integration of neural and biomechanical modeling within NEURON. The models include: (1) a neuromuscular model of calcium dynamics and muscle force, (2) a neuromechanical, closed-loop model of a half-center oscillator coupled to a rudimentary motor system, (3) a closed-loop model of neural control for respiration, (4) a pedagogical model of a non-smooth “brain/body” system, and (5) a closed-loop model of feeding behavior in the sea hare *Aplysia californica* that incorporates biologically-motivated non-smooth dynamics. This tutorial illustrates how NEURON can be integrated with a broad range of neuromechanical models.

**Code available at:**

https://github.com/fietkiewicz/PointerBuilder.

## 1. Introduction

The central nervous system is strongly coupled to the body; through peripheral receptors and effectors, it is also coupled to the constantly changing outside world. The brain, the body, and the environment are each dynamical systems in their own right, and the interactions between them give rise to adaptive behavior (Chiel and Beer, 1997). Therefore, it is important to develop simulation tools that can represent both neuronal dynamics and peripheral biomechanics (Weidel et al., 2016; Falotico et al., 2017).

Currently, there are a variety of platforms for simulating neural systems or biomechanical systems, but relatively few integrate both to simulate neuromechanical systems in a single, cohesive application. The only comprehensive solution in use has been AnimatLab (AnimatLab, 2023). AnimatLab provides the ability to combine neural circuit models, specified by custom-written C++ code, with biomechanical models, implemented using the Vortex physics engine (Cofer et al., 2010). However, to the best of our knowledge, AnimatLab is no longer under active development.

Numerous other solutions have been developed as platforms that combine a dedicated neural simulation application with some other type of software for biomechanical simulation. Other platforms integrating neural and biomechanical dynamics include the Neurorobotics project (Falotico et al., 2017; Feldotto et al., 2022; NRP, 2023) and MUSIC (Djurfeldt et al., 2010; Weidel et al., 2016). NRP is a Linux-based platform that uses the NEST/PyNN neural simulator, the Gazebo physics simulator, and the Robot Operating System (ROS, 2023). The Neurorobotics system is motivated primarily by robotics development, rather than being focused on understanding the neuromechanics of animals. MUSIC was originally developed to facilitate the integration of multiple simulator platforms, and was originally focused on neural simulations (Djurfeldt et al., 2010). It was extended, via ROS and Gazebo, to simulate a Braitenberg vehicle (Weidel et al., 2016), with the goal of using physical robots as tools for studying embodied neural systems.

Notably, while NEURON has been used in combination with other biomechanical simulators, no publicly supported platforms currently exist, to the best of our knowledge. Dura-Bernal et al. developed a cortical spiking network interfaced with a C++ simulation of a virtual musculoskeletal arm and robotic arm (Dura-Bernal et al., 2015, 2016). Moraud et al. implemented a neural network in combination with a model of a rat ankle joint (Moraud et al., 2016) using OpenSIM (Seth et al., 2011). Volk et al. used simulated motoneuron recruitment for control of a model of a human ankle joint implemented with separate finite element modeling software (Volk et al., 2021). However, none of these examples using NEURON have been developed as a publicly supported neuromechanical simulation platform. NEUROiD is a platform that has been used to interface NEURON with OpenSIM (Seth et al., 2011) for simulation of a human ankle joint (Iyengar et al., 2019) and an upper limb (Kapardi et al., 2022). However, NEUROiD is not yet publicly available (personal communication from the author).

In this paper, we describe a method for using the NEURON platform to incorporate biomechanics with the advantage of utilizing a single, cohesive application that does not require advanced software installation. NEURON is a widely used neural simulation platform that has enjoyed continuous development and active support for over three decades (Hines and Carnevale, 1997; Carnevale and Hines, 2006; Hines et al., 2009; Lytton et al., 2016; Awile et al., 2022; McDougal et al., 2022). NEURON is readily extensible and has a large and active user community. A key challenge for integrating neural dynamics and biomechanics is establishing communication between the neural simulator and the biomechanics implementation, in an easy to use programming environment. AnimatLab provides a graphical user interface in which the user can specify a transfer function to map neural variables (firing rates, motor neuron voltages) into mechanical quantities (applied torques, actuator positions), as well as “sensory” functions that map, e.g., muscle fiber activations or distensions to applied currents (Cofer et al., 2010; AnimatLab, 2023). Similarly, in the Neurorobotics platform, communication between NEST/PyNN and Gazebo is accomplished via a “Brain Interface and Body Integrator (BIBI)” that implements a “Transfer Function” framework, that “translates the output of one simulation into a suitable input for the other” (Falotico et al., 2017). In this paper, we take a first step to integrating biomechanics into NEURON, using an existing (but little used) feature of NEURON, namely its pointer architecture.

## 2. Results

We present five models, each of which demonstrates both the philosophy and techniques of a neuromechanical modeling approach using NEURON. The first model demonstrates basic usage of pointers with an example of calcium dynamics and muscle force. The second is a neuromechanical model of a half-center oscillator that contrasts the differences between state variables and parameters. The third model uses the NEURON implicit management of ionic currents with an application for respiratory control. The fourth is a small, elementary model that demonstrates basic techniques for incorporating non-smooth dynamics. The final example incorporates a firing rate model for control of musculature and incorporates a more advanced example of non-smooth dynamics.

### 2.1. Pointers in the NEURON environment: neuromuscular system

In the NEURON environment, a low-level language named NMODL is used to define a *mechanism*, and mechanisms are inserted into a *section* using a high-level language command (Carnevale and Hines, 2006). A program is stored as a plain text file, ending with the extension .mod, and is referred to as a *mod* file. Generally, one NMODL program cannot access the value of a state variable or parameter in another NMODL program without the use of a pointer variable. The only exception to this rule is the use of certain default state variables (e.g., membrane voltage and ion concentrations) and parameters (e.g., resistivity) in the NEURON environment whose values are implicitly shared by all mechanisms in a section. For all others, a modeler must implement connections between variables in different NMODL programs with a *pointer* variable. In this section, we discuss the low-level construction of pointers using NMODL and how a high-level language is used to instantiate the model prior to simulation. We present an example from the perspective of someone beginning to design a model with pointers already in mind. For a discussion of converting an existing mod file that does not already use pointers, see Appendix 1.2 in Supplementary material.

#### 2.1.1. Pointers in NMODL

**Figure 1:**
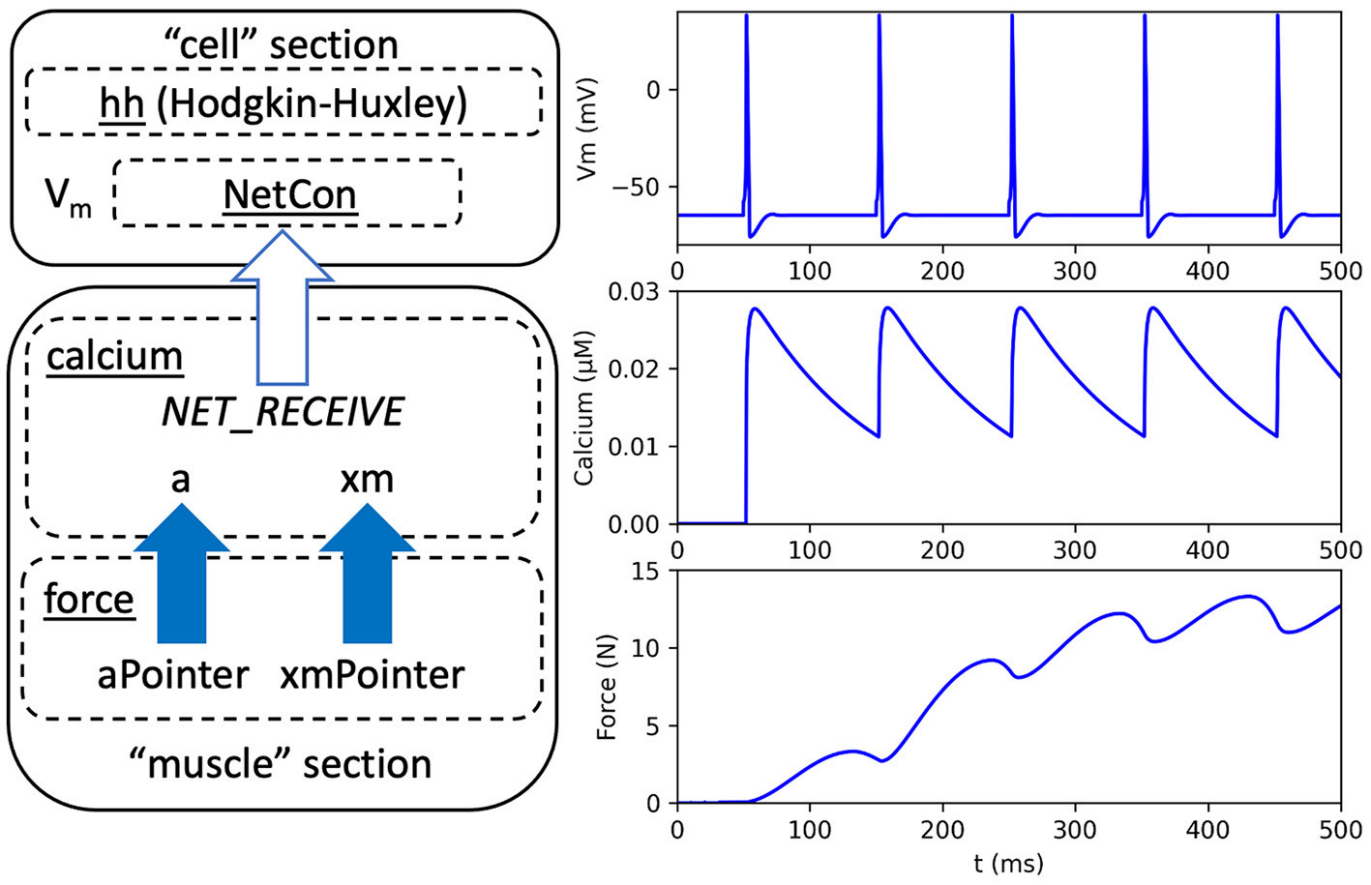
Overview and output of a neuromuscular model adapted from Kim (2020). **Left**: Arrows indicate dependencies between code modules. A neural “cell” section contains a Hodgkin-Huxley mechanism, named *hh*, a network connection mechanism, named *NetCon*, and a membrane voltage (*V*_*m*_). A biomechanical “muscle” section contains a *calcium* mechanism that receives a notification, through *NetCon*, when the membrane voltage exceeds a threshold (see white arrow). The *calcium* mechanism contains variables for activation level (a) and muscle position (xm). A separate *force* mechanism calculates muscle force as a function of muscle activation and position using pointer variables *aPointer* and *xmPointer*, respectively (see solid arrows). **Right**: Model output, using neuron membrane voltage (top), muscle calcium concentration (middle), and force (bottom).

This section demonstrates the use of pointers by adapting a published neuromuscular model by Kim, which is available on (Kim, 2017, 2020; McDougal et al., 2017; Kim and Heckman, 2023). To the best of our knowledge, this model is the only published treatment of muscle dynamics that has been implemented entirely in NEURON. From the Kim model, we use two separate mechanisms for sarcoplasmic calcium dynamics and static muscle force, respectively. Here, we revise the model to show how a previous approach for sharing variables can be replaced with pointers. We also revise the neural components in the Kim model that previously consisted of a multicompartmental reconstruction of a motoneuron with detection of action potentials. As an example of the versatility of NEURON, we instead use standard NEURON library mechanisms, including a Hodgkin-Huxley neuron, named *hh*, and an efficient mechanism for detecting action potentials for network connections, named *NetCon*. An overview of the model is given in Figure 1 that shows the Hodgkin-Huxley neuron, which is new to our adaption, and the mechanisms for calcium dynamics and force calculation, which are modified from the Kim model.

In the remainder of this section, we discuss key techniques for implementing the modular connections shown in Figure 1, and selected program excerpts are given. Interested readers can find the model equations in Appendix 1.1 (Supplementary material) and complete program files using the link provided in Section 4. In our adaptation of Kim's model, the *force* mechanism uses pointers to access the variables for muscle activation (*a*) and muscle length (*xm*) that are located in the *calcium* mechanism. The pointer variables are declared in the NEURON block, as shown in the NMODL example below, and they can be used throughout the program.


       :: NMODL code ::      NEURON {          POINT_PROCESS force          POINTER aPointer, xmPointer      } 

As shown in Figure 1, the *calcium* mechanism depends on the membrane voltage of the neuron. When an action potential occurs in the neuron, the *calcium* mechanism computes the postsynaptic release of Ca^2+^, as detailed in Appendix 1.1 (Supplementary material). In our adaptation of Kim's model, we apply a mechanism that is widely used for synaptic connections in NEURON, called *NetCon*, which is part of the standard library (Hines and Carnevale, 2004). The NetCon mechanism efficiently notifies dependent mechanisms when a variable exceeds a given threshold, such as when a membrane voltage exhibits an action potential. In neuronal networks, a NetCon can be used in combination with a synapse to prevent the need for receivers to constantly monitor the source by themselves. For a receiver mechanism to respond, it must include a NET_RECEIVE function that is called by the source mechanism and executed when an event occurs.

**Figure 2:**
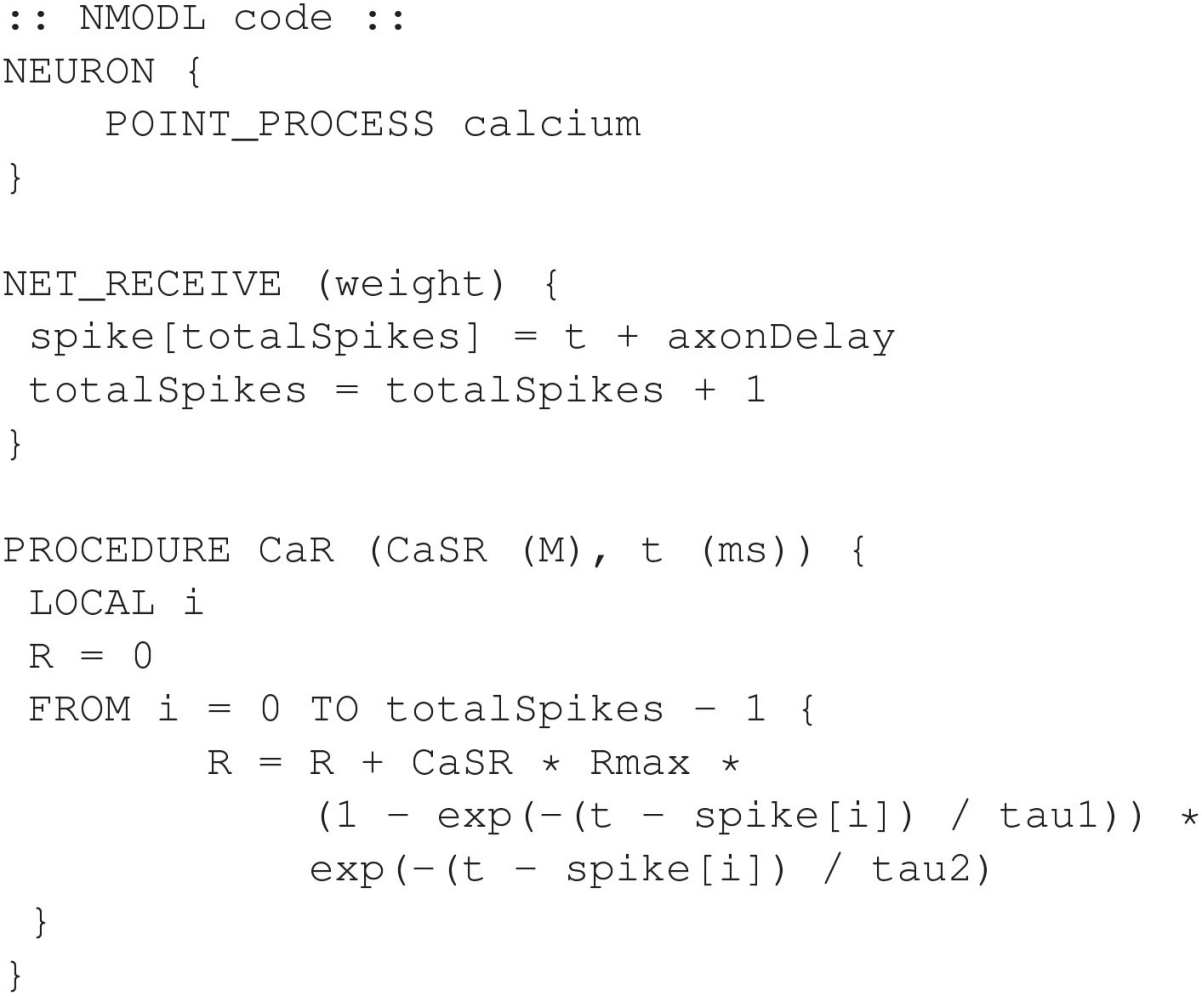
Excerpts from calcium.mod. The NET_RECEIVE function efficiently tracks action potentials in the neuron. The CaR function computes the calcium release for use in the BREAKPOINT block (not shown). The complete program is available in the public code repository.

In the original Kim model, the *calcium* mechanism requires knowledge of the previous times at which the neuron had action potentials, or spikes. These spike times were stored in an array and then used to calculate the release of calcium, as detailed in Appendix 1.1 (Supplementary material). Our adaption uses a NET_RECEIVE function to update the array of past spike times only when a *NetCon* mechanism detects an action potential. Figure 2 shows an excerpt from the *calcium* mechanism that shows the NET_RECEIVE function and a subroutine named CaR that, as in the Kim model, computes the calcium release (*R*).

The complete program file for the *calcium* mechanism is available using the link provided in Section 4.

#### 2.1.2. Connecting pointers for state variables using the “hoc” language

Simulation of mechanisms requires one of the two high-level languages that are supported in NEURON. One language is known as hoc (pronounced with a long “o” sound, as in “spoke”), and the other is Python. We first discuss using the hoc language because it was initially the only high-level language supported in NEURON. For numerical integration of the differential equations, hoc instructions are used to insert a mechanism into a *section*. A section is traditionally used to represent a part of a neuron, such as a soma, an axon, or a dendrite. However, a section is just a programming object that may have its own unique assortment of mechanisms and properties. Therefore, a section is a required construct for numerical integration of differential equations in NEURON.

In the following hoc instructions, a NEURON section named *cell* is used for inserting the built-in *hh* mechanism, and a section named *muscle* is used for inserting mechanisms *calcium* and *force*.


       // hoc code //      // Create neuron model      create cell      cell {        insert hh      }        // Create muscle model      objref calciumObject, forceObject        create muscle      muscle {          calciumObject = new calcium(0.5)          forceObject = new force(0.5)      } 

**Figure 3:**
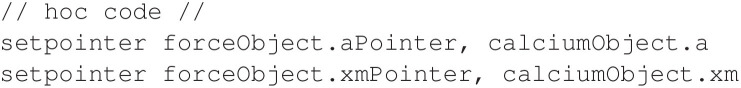
Configuring pointers for the neuromuscular model.

The final technique to be discussed is the hoc instruction *setpointer* that is used to configure each pointer variable. Pointer dependencies are configured in hoc using the following syntax:

setpointer *pointer*,*original*

where *pointer* is a pointer variable that has been declared in a POINTER statement in an NMODL program, and *original* is the original variable being referenced, being either a state variable or parameter. In the setpointer syntax given above, *pointer* and a state variable *original* must both use the following syntax:

*section.variable*

where *section* is the section in which the mechanism has been inserted, and *variable* is the name of the pointer variable or state variable. For the example model, Figure 3 shows the hoc statements that will configure the necessary pointers.

**Figure 4:**
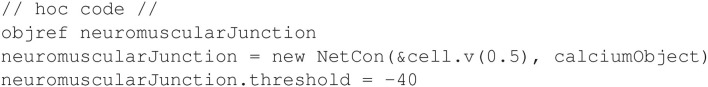
Configuring the NetCon for the neuromuscular model.

In Figure 3, *aPointer* and *xmPointer* are pointer variables in the *force* mechanism that will access the state variables *a* and *xm*, respectively, in the *calcium* mechanism. To complete the model, a *NetCon* mechanism is created to emulate a neuromuscular junction and notify the *calcium* mechanism when an action potential occurs in the neuron. The code in Figure 4 creates and configures the *NetCon*.

In the code above, the *neuromuscularJunction* object will monitor the membrane voltage (*v*) of the *cell* section. When the voltage reaches a value of −40 mV or higher, the NET_RECEIVE function of the *calcium* mechanism will be executed.

Using the full model, Figure 1 shows the output, including neuron action potentials, calcium concentration, and muscle force. Note that the Kim model (Kim, 2017, 2020), which served as a basis for the model used here, achieved a modular programming design without using pointers. Appendix 1.1 in Supplementary material discusses differences between Kim's implementation and ours.

The example above demonstrates the basic technique of using pointers. It is valuable to consider the potential effect that using pointers has on numerical stability in the simulation. If all differential equations were in the same file, with no pointers, NEURON would construct a Jacobian with all the appropriate cross-terms that would completely couple all the different components. Using pointers creates a Jacobian without all of the cross-terms, and this could induce instability. Such instability can be mitigated by using smaller timesteps. When using NEURON's variable time step solver, NEURON will do this automatically, in that it will choose timesteps that let the Jacobian be “good enough”. Nevertheless, it is always important for the user to empirically check for convergence by halving the time step and confirming that the results are essentially the same.

### 2.2. Connecting pointers for parameters: half-center oscillator system

In the previous section, pointers were used to access state variables. Pointers can also be used to access parameter variables, whose values can be changed after an NMODL program has already been compiled. In general, a parameter variable would likely only need to be accessed within a single NMODL program, and a pointer would not be necessary. However, a pointer to a parameter could be of use in special circumstances, such as using a single parameter across multiple NMODL programs or any other situation where use of a state variable is undesirable. The following section discusses the syntax for pointers to parameters and presents a model that demonstrates this technique.

To understand pointers to parameters, one must first be aware of the different ways that variables are categorized in NMODL. Variables are first categorized according to how they are declared in the program, which determines how they are used. This includes, but is not limited to, categories such as STATE, PARAMETER, and POINTER, of which the latter allows access to the other variables. A secondary way to categorize variables is with regard to how they are handled in multicompartment simulations, where each variable is either a *range* or *global* variable.

The category of range variables includes any variable whose value is dynamically calculated as a function of the discretized position within a multicompartmental section. By default, all state variables are considered range variables, but parameter variables are not. The hoc syntax for pointers to parameter variables depends on whether the parameter is configured in the RANGE block. If the modeler specifically configures a parameter as a range variable, the syntax explained previously for pointers would still apply. In the following example we discuss the default case where a pointer is used for a parameter that is not also a range variable.

The general format for the setpointer instruction, as introduced in the previous section, still applies here:

setpointer *pointer*,*original*

where *pointer* is a pointer variable that has been declared in a POINTER statement in an NMODL program, and *original* is a parameter. However, a pointer to a parameter with default characteristics (i.e., not a range variable) requires a different syntax for the term *original*, as compared to the previous example that used only state variables. For a parameter, the term *original* contains only the parameter name and the mechanism name. The section name and physical location are omitted because the parameter value applies uniformly to all segments. In this case, the term *original* would take the following form:

*parameter* _ *mechanism*

where *parameter* is the default parameter variable that is defined in *mechanism*.

To demonstrate the use of pointers to parameter variables, we use a model for motor pattern generation that combines central pattern generator dynamics with sensory feedback (Yu and Thomas, 2021). The model equations are reproduced in Appendix 1.4 (Supplementary material). In the model, membrane voltages *V*_1_ and *V*_2_ depend on muscle lengths *L*_1_ and *L*_2_, respectively; and muscle activations *A*_1_ and *A*_2_ depend on membrane voltages *V*_1_ and *V*_2_, respectively.

The NEURON implementation is comprised of separate *brain* and *body* mechanisms. The *brain* mechanism defines differential equations for the membrane voltages. The *body* mechanism defines differential equations for the muscle activations, as well as equations for the muscle lengths. Of special importance is that muscle lengths *L*_1_ and *L*_2_ are not defined by differential equations and do not need to be treated as state variables. We can make *L*_1_ and *L*_2_ accessible to the *brain* mechanism by declaring them in the *body* mechanism as parameter variables.

**Figure 5:**
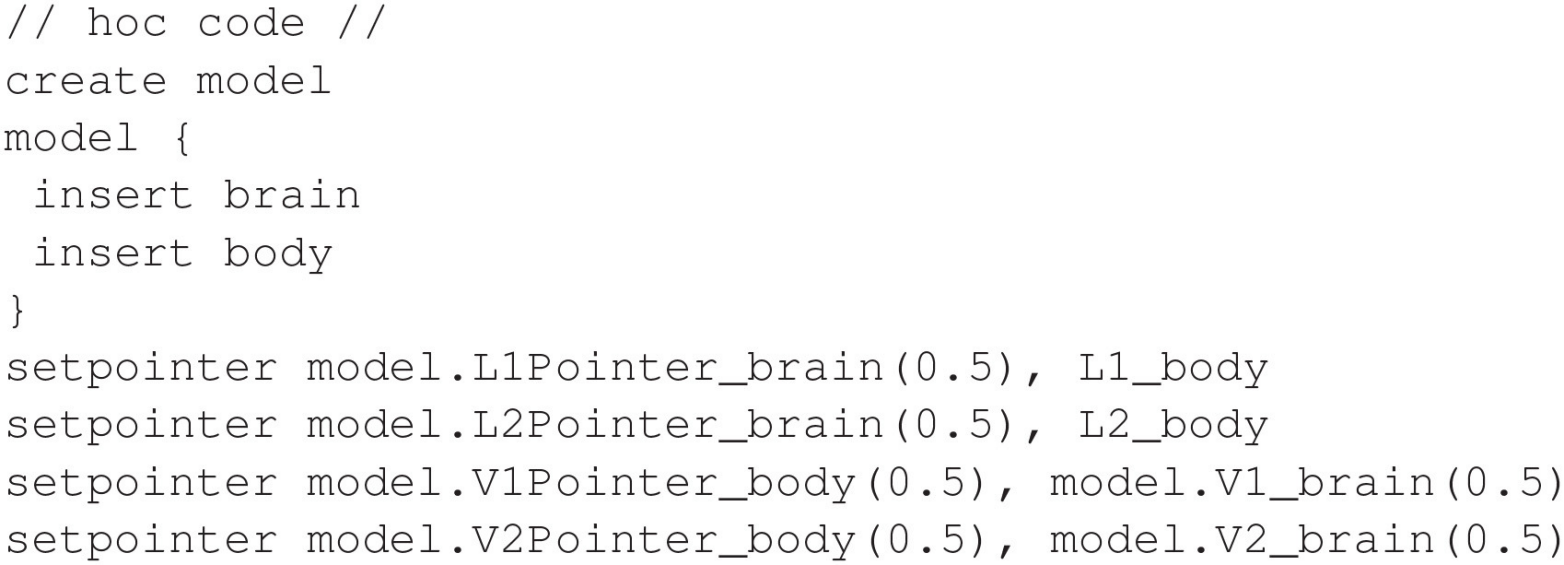
Configuring pointers for the half-center oscillator model.

The *body* mechanism declares parameters L1 and L2, and the *brain* mechanism declares pointers L1Pointer and L2Pointer (see Section 4 for a link to the full programs). Finally, a hoc program connects the pointers, as shown in Figure 5.

**Figure 6:**
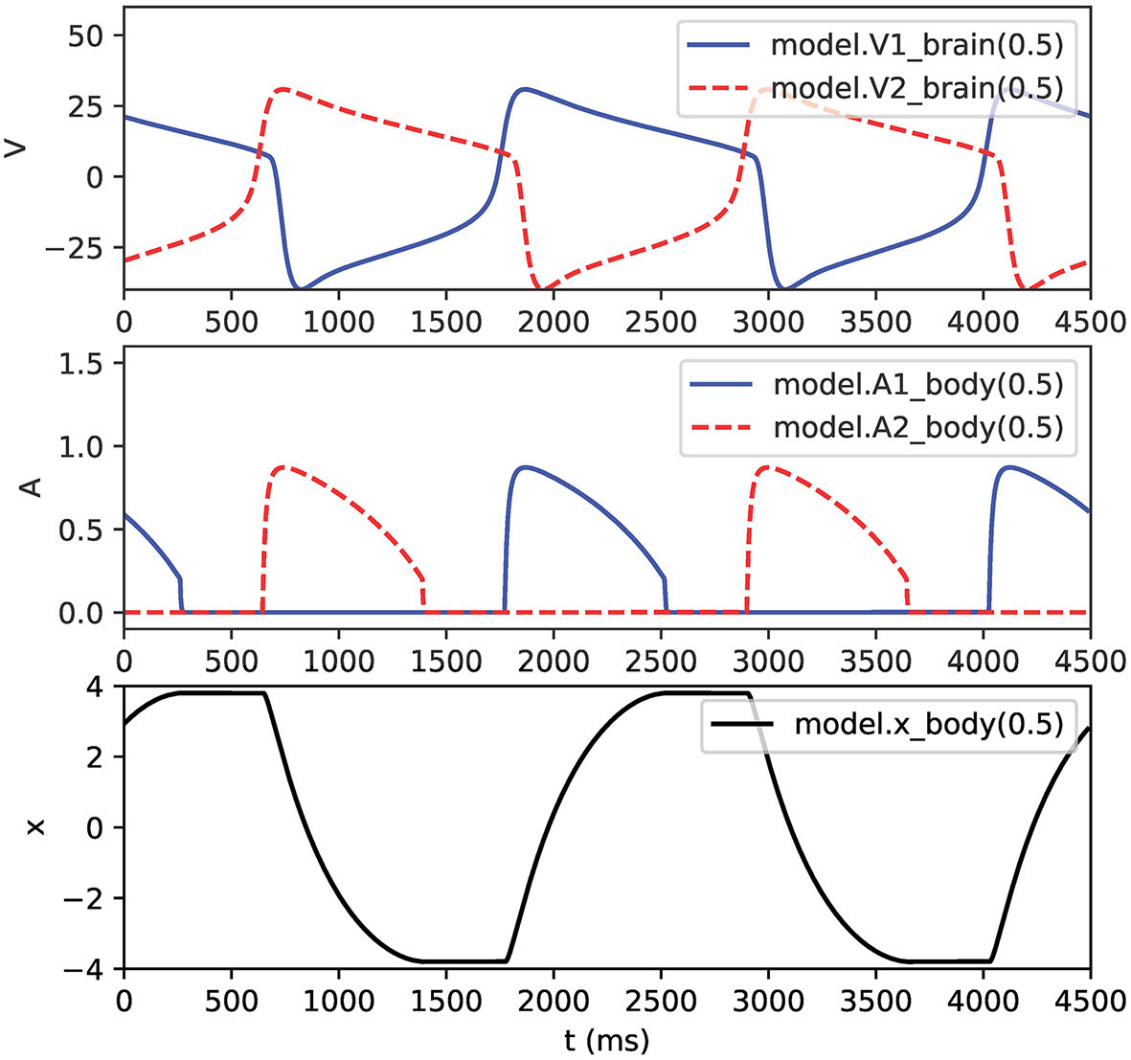
Output of half-center oscillator model. **Top**: membrane voltages *V*_1_ and *V*_2_. Middle: muscle activations *A*_1_ and *A*_2_. **Bottom**: pendulum position *x*. Legends show hoc syntax used for graphing the variables.

Note that the example in Figure 5 also connects pointers from the *body* mechanism to the membrane voltages V1 and V2 in the *brain* mechanism. Figure 6 shows the output of this NEURON implementation.

### 2.3. Connecting pointers using Python: neuromuscular model revisited

NEURON provides a programming interface for the Python language. While NEURON runs independently, the Python interface allows one to control and analyze NEURON simulations using Python programs and packages, such as machine learning tools (see Hines et al., 2009; Gratiy et al., 2018).

Python syntax is significantly different than hoc syntax, and a thorough discussion is beyond the scope of this paper. Briefly, the NEURON-Python interface is based on an object-oriented approach, and NEURON functionality begins with a primary object named “h”.

In this section, we revisit our reimplementation of Kim's neuromuscular model and present a Python implementation that is equivalent to the hoc version presented earlier. The NMODL programs *calcium.mod* and *force.mod* are unchanged, and they can be inserted into a section as follows.


       ## Python code ##      body = h.Section(name = 'body')      calciumObject = h.calcium(body(0.5))      forceObject = h.force(body(0.5)) 

After creating a section and inserting mechanisms, there are two different ways to configure pointers using Python. One way uses a direct, object-oriented syntax and is the recommended approach. The second way uses the Python *setpointer* function, similar to the approach used with hoc that was presented in Section 2.1. Both approaches are equivalent and given for completeness.

The recommended way to configure pointers is to use an assignment statement with the “=” operator and an object-oriented syntax. Note that the ordering of the terms is consistent with the ordering for the hoc syntax, whereas the Python setpointer function (described later) uses a different ordering that may be confusing when compared to hoc. The basic object-oriented syntax is as follows:

*pointer* = *original*

where *pointer* is a pointer variable that has been declared in a POINTER statement in an NMODL program, and *original* is either a state variable or parameter. In the assignment statement given above, *pointer* and a state variable *original* can both use the following syntax:

*section* ( *position* ). *mechanism* ._ref_ *variable*

where *section* is the section in which *mechanism* has been inserted, *position*∈[0, 1] is the relative physical position of the variable in the section, the prefix “_ref_” indicates a reference to a variable, and *variable* is the name of the pointer variable or state variable.

**Figure 7:**
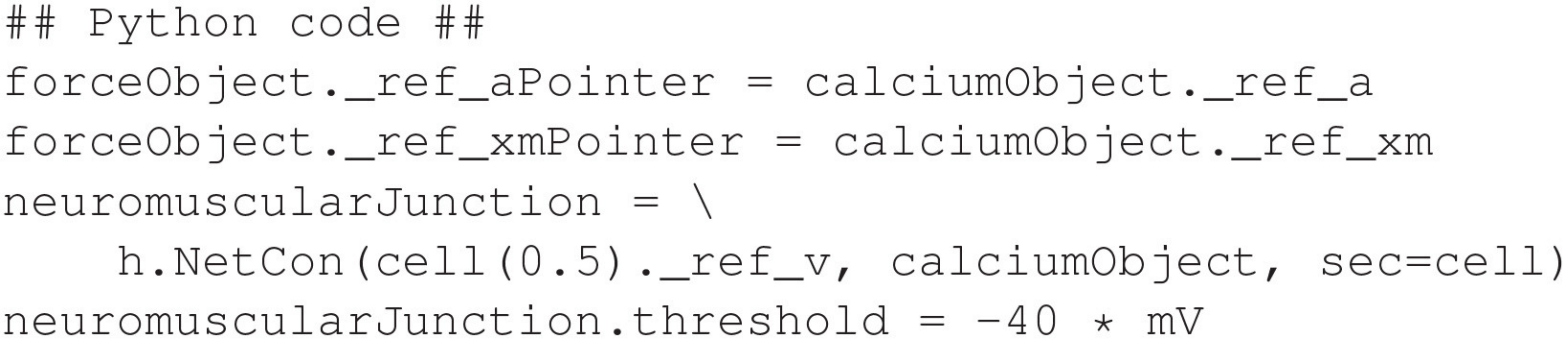
Python is used to configure pointers in the neuromuscular model, as well as create the NetCon instance.

**Figure 8:**
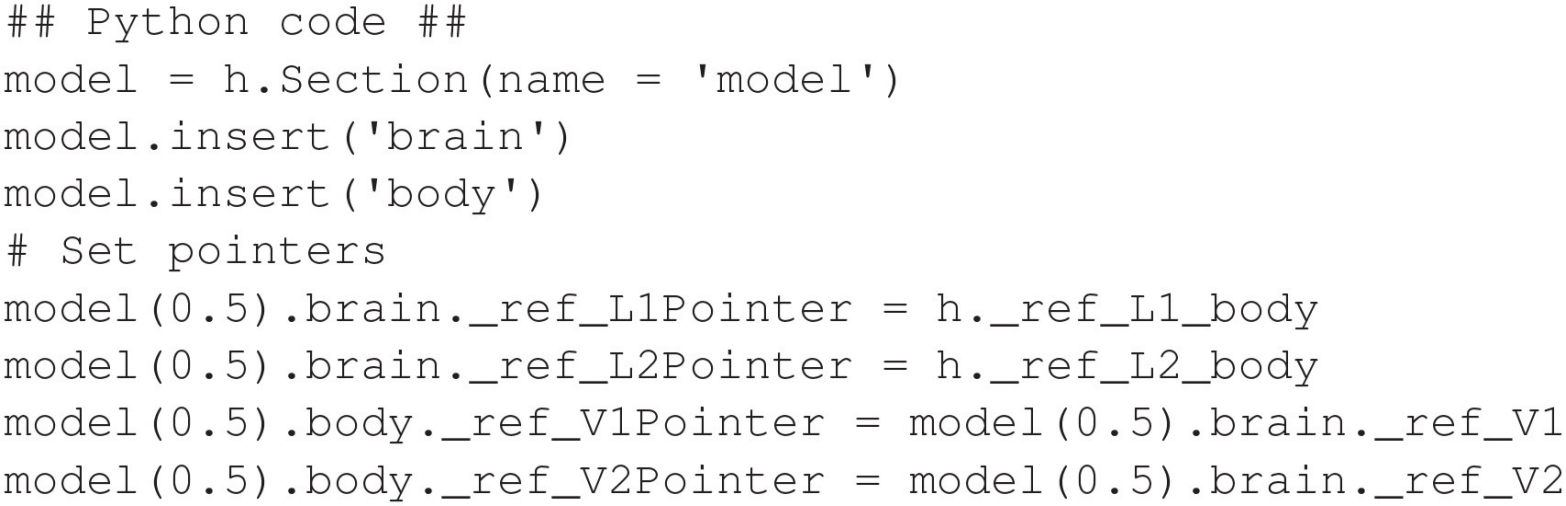
Python is used to configure pointers in the half-center oscillator model.

For the neuromuscular model, Figure 7 shows the Python statements that would configure the pointers, as well as create the *NetCon* for the dependency of the *calcium* mechanism on the neuron voltage:

Recall that the syntax for pointers to parameter variables is different than that for state variables. As explained in Section 2.2, the section name is omitted when referencing a parameter. Figure 8 demonstrates the Python equivalent for the half-center CPG model in Section 2.2, where the variables L1 and L2 are parameters in the *body* mechanism.

For completeness, the following presents an additional way to configure pointers in Python. In contrast to the approach discussed above, a *setpointer* function is also available, though it is more complicated to use than the assignment technique above. Using the Python *setpointer* function is similar to the approach used with hoc that was presented in Section 2.1, with two exceptions.

**Figure 9:**
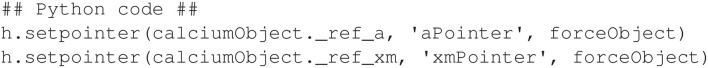
Alternative Python syntax to configure pointers in the neuromuscular model.

First, the order of the two variables is reversed; and second, the name of the pointer variable is given as a separate argument to the setpointer function. The setpointer function uses the following syntax:

h.setpointer(*original*, *pointer name*, *pointer mechanism*)

where *original* is either a state variable or parameter, *pointer name* is a pointer variable that has been declared in a POINTER statement in an NMODL program, and *pointer mechanism* is the mechanism that contains the pointer. Figure 9 demonstrates the alternative setpointer instructions necessary to connect the pointers in the neuromuscular model described at the beginning of this section.

Note that Appendix 1.3 (Supplementary material) discusses an application called PointerBuilder that can be used to learn and verify pointer syntax in the Python language.

### 2.4. Implicit current management in NEURON: closed-loop respiratory model

A distinctive feature of NEURON is its ability to implicitly manage ion currents across a capacitive cell membrane. When using this feature, the membrane voltage equation can be computed without the need for the modeler to specify a differential equation. Additionally, transmembrane currents are automatically summed, allowing each mechanism to specify its own contribution to a particular current. This allows a modeler to easily maintain an independent NMODL program for each current, if desired. Here we present a model that uses the implicit current management feature in NEURON. The model originally appeared in Diekman et al. (2017) using a different simulation platform. It is a closed loop system comprised of a respiratory control neuron (Butera et al., 1999), together with lung mechanics, oxygen handling, and chemosensation (Hlastala and Berger, 2001; West, 2008; Keener and Sneyd, 2009; Ermentrout and Terman, 2010).

The NEURON implementation presented here uses the current management feature extensively by defining a separate mechanism for each of the following transmembrane currents: potassium, sodium, persistent sodium, leak, and synaptic. The full model, including equations, is detailed in Appendix 1.5 (Supplementary material). It uses pointers in two different contexts. First, pointers are used to connect neural mechanisms with other physical mechanisms in the closed-loop system, similar to the models in previous sections. Specifically, neural activity drives a motor response, and chemosensation produces synaptic input to the neuron. Second, a pointer is used to support a dependency between the equations for the sodium and potassium currents such that each current can be defined in a separate NMODL program (see Appendix 1.5 in Supplementary material). The following discusses the pointer configuration and the use of NEURON's implicit current management.

Similar to the approach of models in previous sections, all non-neural components are implemented in a single NMODL mechanism. This includes lung mechanics, oxygen handling, and chemosensation, and these are contained in a single mechanism named *respiration*. Note that the non-neural components could also have been separated through the use of pointers, but here we focus on using pointers only for connections between neural and peripheral components. Specifically, respiratory muscle activity depends on the membrane voltage *V* of the neuron, and synaptic current in the neuron depends on a synaptic conductance *g*_*tonic*_ that is computed from the partial pressure of oxygen in the lung alveoli (*P*_*a*_*O*_2_).

**Figure 10:**
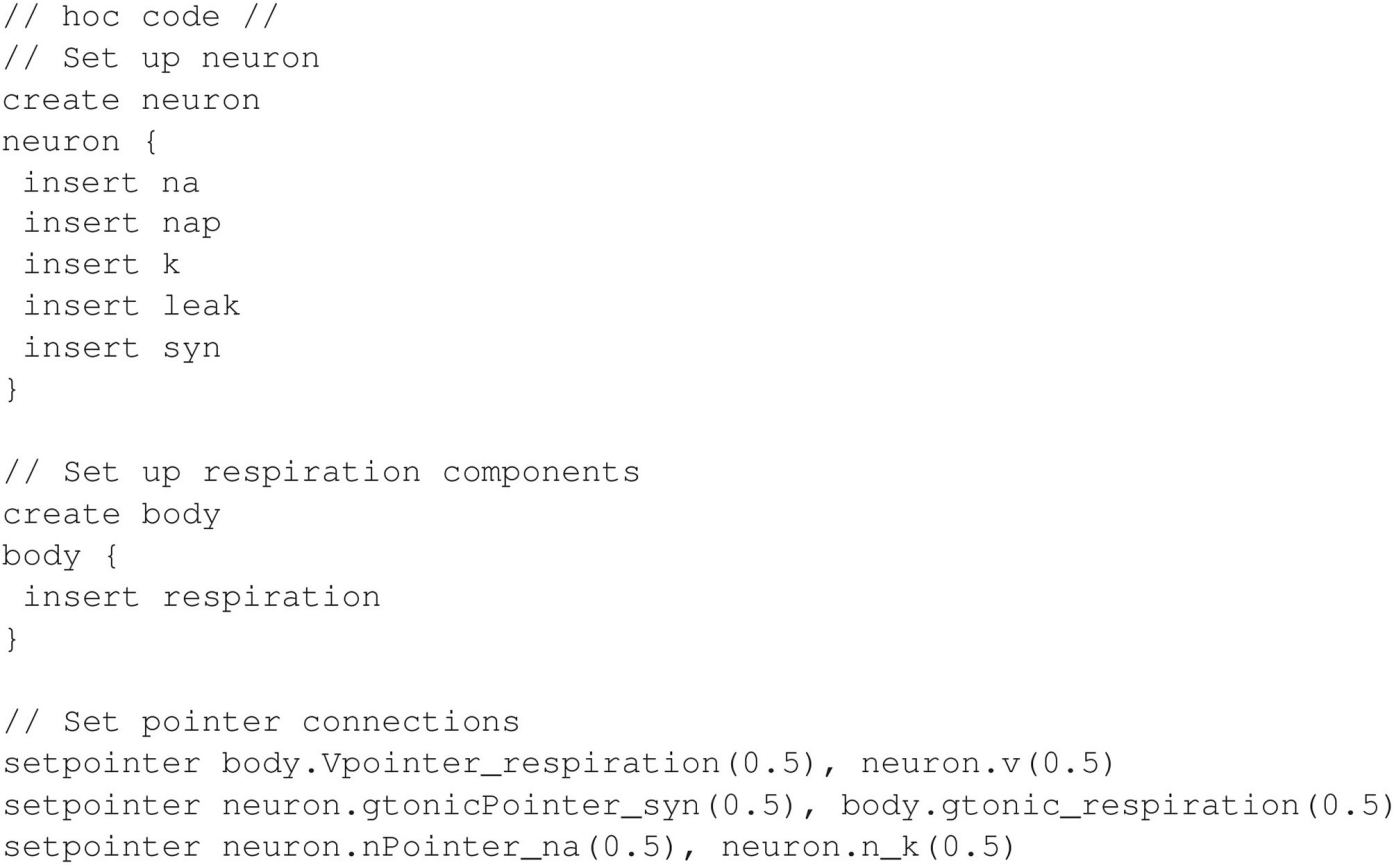
Inserting mechanisms and configuring pointers for closed-loop respiratory model.

As mentioned earlier, each transmembrane current in the neuron is defined in a separate mechanism. Additionally, sodium inactivation depends on the potassium variable *n*, which represents a delayed-rectifying activation (see Appendix 1.5 in Supplementary material). This dependency requires a third pointer in the model. In total, there are five mechanisms for the transmembrane currents: *k* for potassium, *na* for a fast sodium, *nap* for a persistent sodium, *leak* for a leak current, and *syn* for the synapse. All of the neural mechanisms are inserted into a section named *neuron*, and the *respiration* mechanism is inserted into a separate section named *body*. The hoc code shown in Figure 10 inserts the necessary mechanisms and connects the pointers.

**Figure 11:**
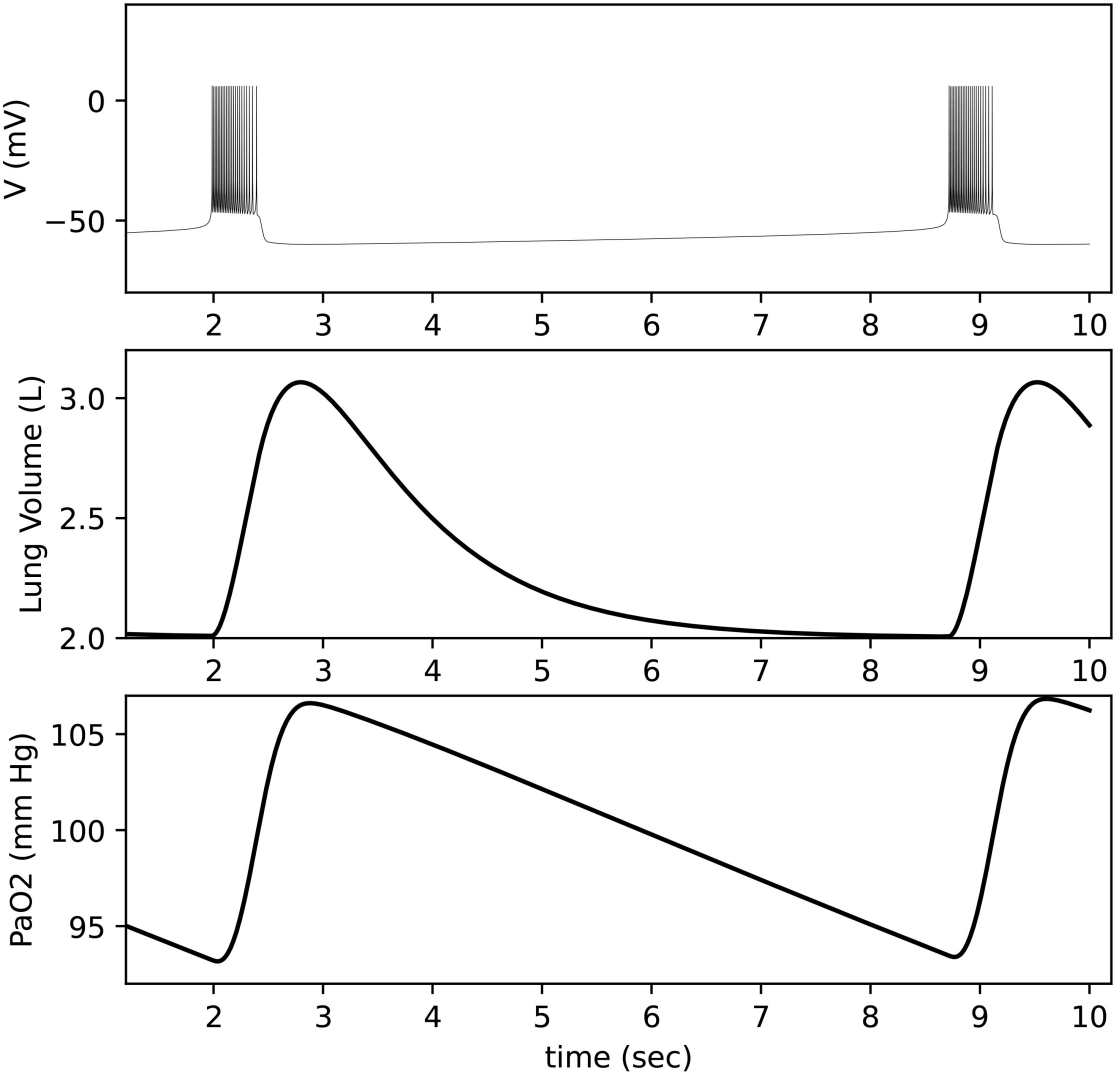
Closed-loop respiratory model output. **Top**: Neuron membrane voltage shows a bursting pattern. **Middle**: Lung volume is calculated based on activity from a motor pool that is driven by the neuron. **Bottom**: Blood oxygen (*P*_*a*_*O*_2_) is calculated based on lung volume and lung oxygen (not shown).

**Figure 12:**
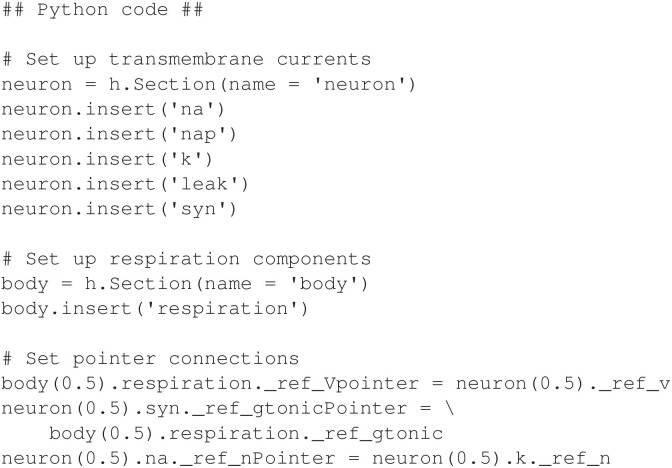
Python code for inserting mechanisms and configuring pointers for closed-loop respiratory model.

The model output is shown in Figure 11. For comparison, Figure 12 shows the equivalent Python code.

### 2.5. Non-smooth dynamics (basic): oscillator model

Modeling of biomechanics often requires the representation of non-smooth dynamics in which the rate of change of certain variables changes discontinuously along boundaries defining subregions of the variable domain. For example, when part of the body makes contact with an external substrate, typically one or more degrees of freedom are removed from the system, and are restored when the body breaks contact with the substrate. Then, the values of one or more variables may be constrained to lie in a prescribed subset during part of their trajectory. This situation arises during the swing-stance transition in locomotion (Spardy et al., 2011a,b) and when the grasper engages or releases a strip of seaweed in the sea hare *Aplysia californica* (Shaw et al., 2015; Lyttle et al., 2017). Incorporating non-smooth dynamics into neuromechanical simulations requires careful implementation. As an example of how to handle this situation in the NEURON context, we consider a simplified “firing rate model” comprising two variables. A scalar firing rate *a*(*t*)≥0 obeys “excitable” dynamics given by the logistic equation


$$\frac{da}{dt}=f\left(a,b\right)=a\left(1-a\right)-b.$$

Here we consider the “body” variable *b* to represent an external drive—inhibitory if *b*>0—that is periodically driving the firing rate up and down, say as


$$\frac{db}{dt}=-b_{0}\omega \sin\left(\omega t\right).$$

However, the firing rate cannot become negative, so we must supplement these equations with a hard boundary condition at *a* = 0. Thus, in effect, the variable *a* follows separate differential equations depending on whether the constraint is active or not, resulting in an example of a non-smooth or Filippov system (Filippov, 1988; Jeffrey, 2020). When *a*>0 the rate of change is given by the equation above. When *a* = 0, then if the input *b*>0 would drive *a* outside the allowed region, the rate of change of *a* must simply remain equal to zero. However, if the input *b* ≤ 0 would not force *a* beyond the constraint, then *a* is allowed to follow the previous ODE. Formally, the condition may be written as follows:


(1) $$\frac{da}{dt}=\{\begin{array}{ll} a(1-a)-b, & \text{ if }(a>0)\text{ or }(a(1-a)-b\geq 0)\\ 0, & \text{ otherwise } \end{array}$$

(2) $$\begin{array}{ll} \frac{db}{dt} & =-b_{0}\omega \sin\left(\omega t\right) \end{array}$$

This simple model provides another example of using pointers. To implement it, we declare state variables *a* and *b* in separate NEURON mechanisms. In the following, we present three techniques for programming logical conditions in the model, like those in Equation (1). The methods vary in their conceptual transparency, their compatibility with variable time-step integration, and their potential appeal to different user communities. These techniques do not require the use of pointers, but they are relevant in the context of neuromechanical modeling where pointers may be used.

The first technique is to use a logical *if* statement in the BREAKPOINT block. Though Equation (1) defines the behavior of the differential equation, it happens that the simplest approach, programmatically, is to control the value of the state variable itself, instead of the differential equation (It will be shown later that using an *if* statement for the differential equation is more complicated.).

**Figure 13:**
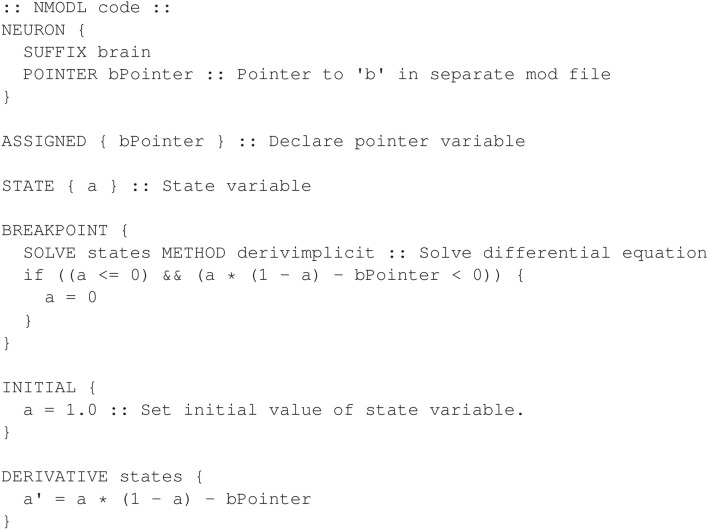
Defining the *brain* mechanism for the non-smooth oscillator model. The logic is performed in the BREAKPOINT block.

It can be observed that Equation (1) contains conditional logic. To achieve this logic, we first use the SOLVE statement, which executes the DERIVATIVE block named “states”, to evaluate the non-zero differential equation in Equation (1) as if the first condition were true, without the use of an *if* statement. Following the SOLVE statement, an *if* statement is used to reset *a* to zero if the second condition in Equation (1) is found to be true. The NMODL program in Figure 13 defines a *brain* mechanism with this logic in the BREAKPOINT block.

**Figure 14:**
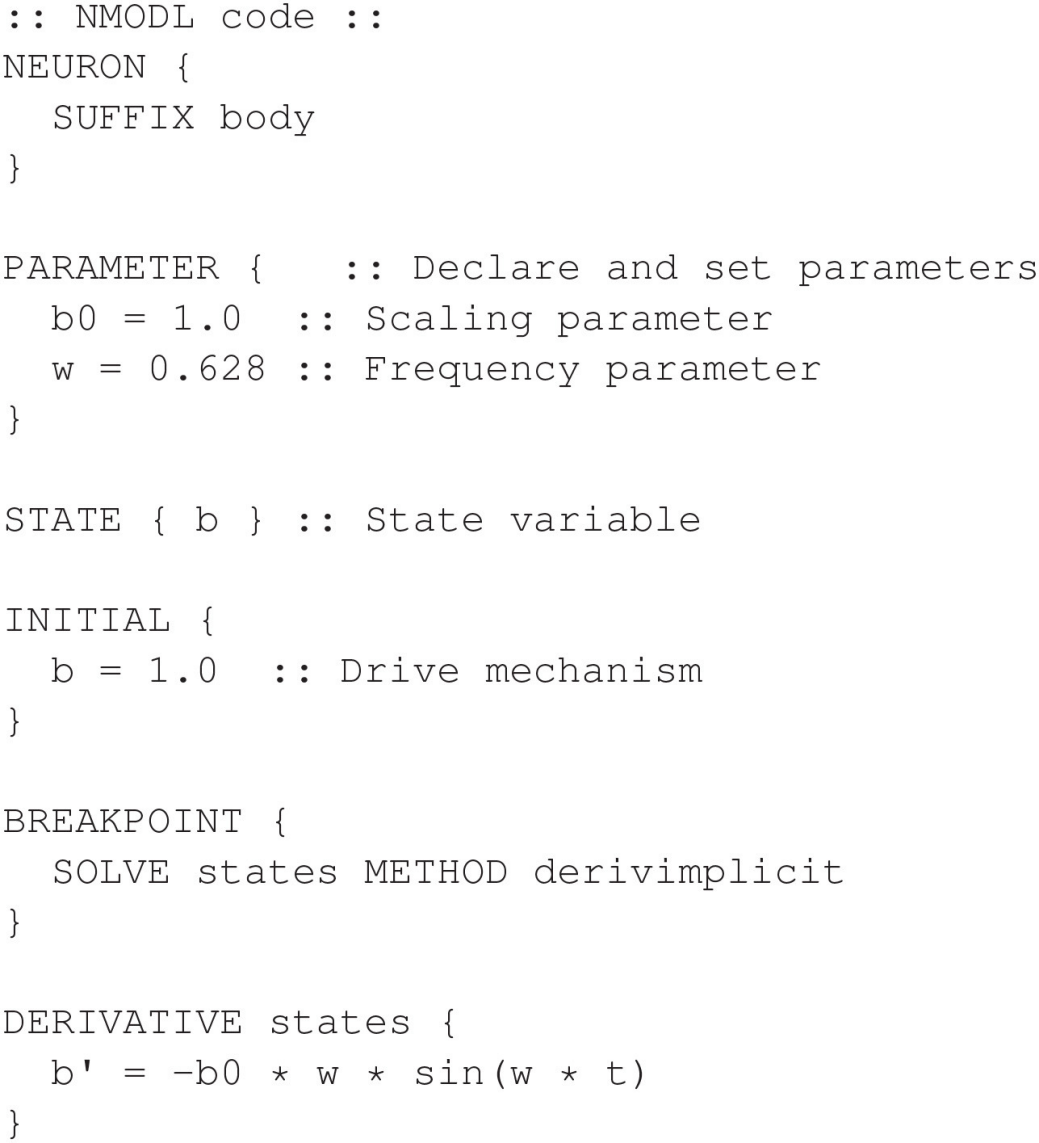
Defining the *body* mechanism for the non-smooth oscillator model.

Figure 14 shows the corresponding NMODL program for the *body* mechanism that declares the state variable *b* and parameters *b*_0_ and ω.

**Figure 15:**
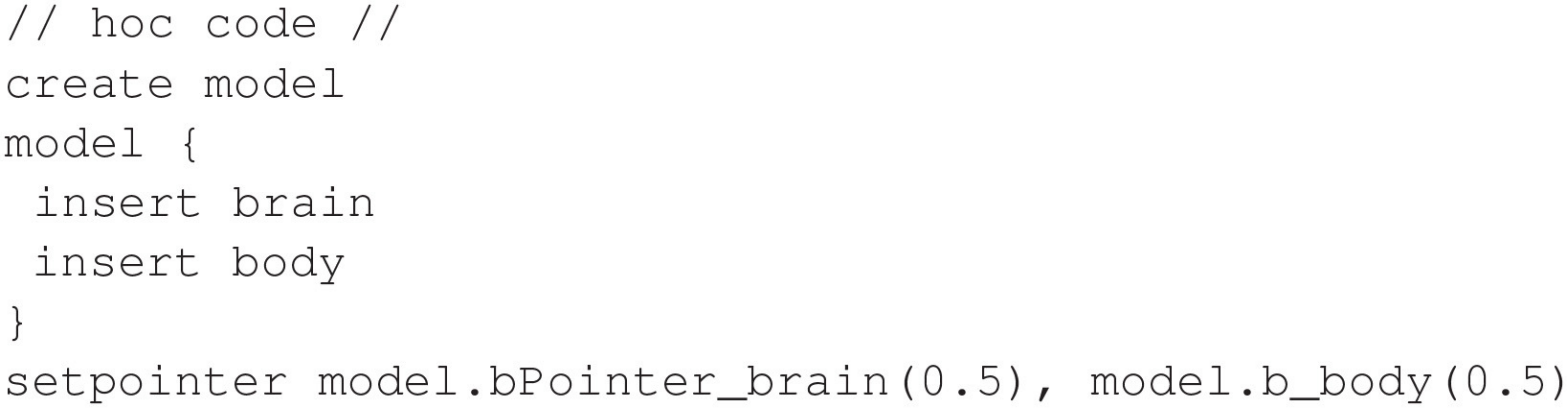
Creating a section and configuring the pointer for the non-smooth oscillator model.

**Figure 16:**
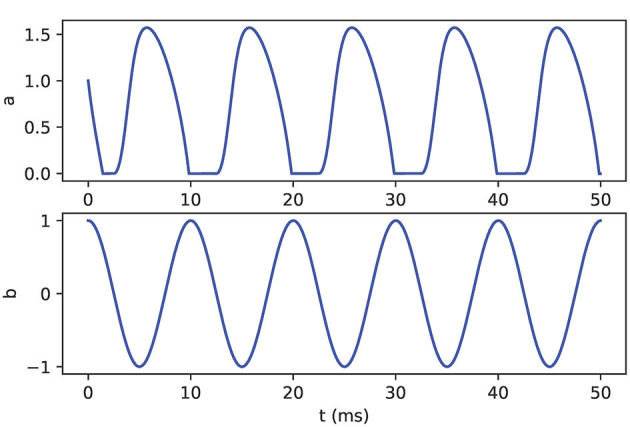
Oscillator model output. The variable *a* **(top)** is driven by the oscillatory variable *b* **(bottom)**. Variable *a* demonstrates non-smooth dynamics in which the value is limited to non-negative values.

Using the above mechanisms, the hoc program shown in Figure 15 shows how to create a section and connect the pointer. Figure 16 shows the output of the full model.

**Figure 17:**
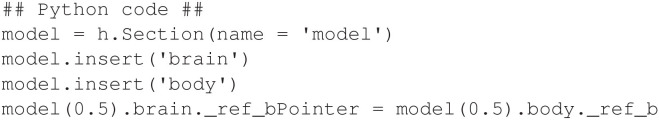
Python code for creating a section and configuring the pointer for the non-smooth oscillator model.

For comparison, Figure 17 shows the equivalent Python code.

Next, a second technique is used to address a limitation with the technique described above. Though the previous technique is the least complicated with regard to programming, it is not compatible with the NEURON feature of *variable time step integration*, where the integration time step can be dynamically adjusted during the simulation for efficiency. The use of variable time step may significantly improve computational speed by dynamically choosing larger time steps, when doing so would not reduce accuracy. This feature can be enabled programmatically or through the standard NEURON graphical interface, and the modeler can specify the level of integration accuracy.

The incompatibility of the previous technique is due to the way in which variable time step integration reevaluates derivatives during the simulation. At each integration step, derivatives are computed for multiple time step values to determine the largest step size that can maintain the minimum accuracy. However, the forced resetting within the BREAKPOINT block disrupts the ability of the algorithm to compare different step sizes.

In the second technique, the incompatibility is mitigated by performing conditional logic at the level of the DERIVATIVE block instead of the BREAKPOINT block. In this case, the logic more closely resembles the original logic given in (1). The disadvantage to this approach is that it requires a more complex program design.

**Figure 18:**
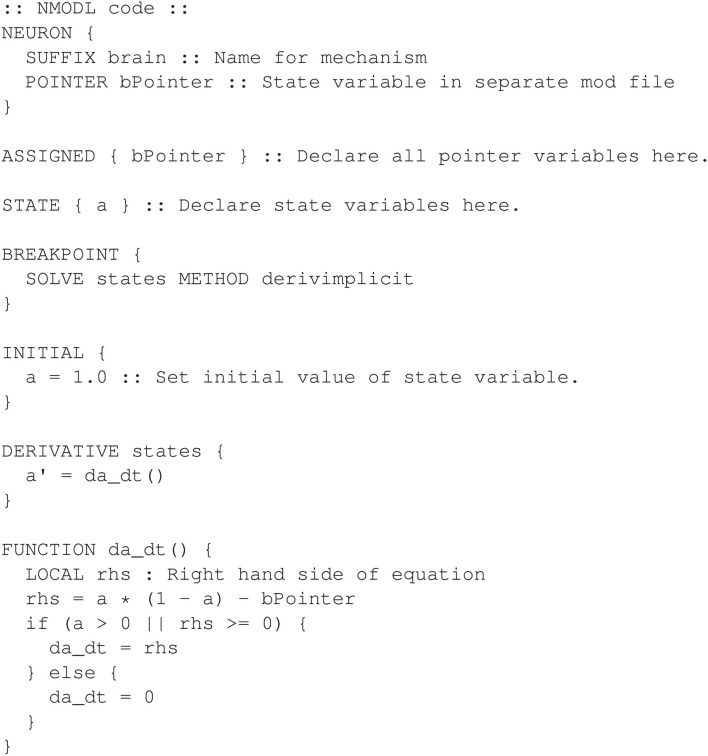
Alternative design for the non-smooth oscillator model. Logic is performed in the DERIVATIVE block through the use of the FUNCTION da_dt.

To implement Equation (1), a particular restriction in the NMODL language must be considered. The DERIVATIVE block is restricted to defining equations using single programming statements. This allows the compiler to accurately parse the syntax for the differential equations. Consequently, additional programming statements, such as *if* statements, cannot be added directly to the DERIVATIVE block. Instead, additional programming statements can be accommodated by defining a separate FUNCTION block which is called directly by the differential equation statement in the DERIVATIVE block. In our example, the right hand side of Equation (1) is implemented in a FUNCTION block. The NMODL program for the *brain* mechanism is given in Figure 18 and uses a function named *da_dt()* to calculate the right hand side of the differential equation.

With the above revisions, the new oscillator model can now be simulated after enabling variable time step integration through either the *VariableStepControl* tool, available through the NEURON graphical interface, or programmatically. When doing so, one can specify a minimum integration accuracy. We compared run times with and without variable time step and found a 28.6% improvement when using variable time step (see Section 4 for methods used).

Finally, we explain a third technique for implementing non-smooth dynamics that is also compatible with the use of variable time step integration. This technique uses Boolean comparisons for the differential equations without an explicit if/else statement. Instead, the Boolean comparisons are incorporated into numeric expressions to achieve the same effect. The technique works because Boolean comparisons in the NMODL language return 1 for *true* and 0 for *false*. Therefore, we modify Equation 1 as follows:


(3) $$\begin{array}{ll} f\left(a,b\right) & =a\left(1-a\right)-b \end{array}$$

(4) $$\begin{array}{ll} \frac{da}{dt} & =\left(a>0\right)f\left(a,b\right)+\left(a\leq 0\right)\left(f\left(a,b\right)\geq 0\right)f\left(a,b\right) \end{array}$$

**Figure 19:**
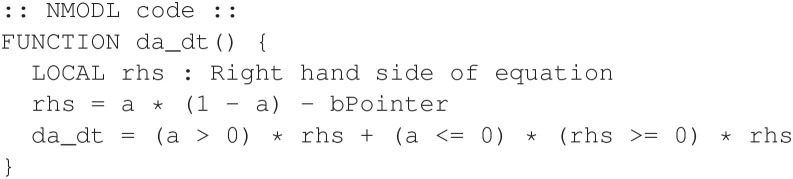
Alternative design for the non-smooth oscillator model. Logic is performed algebraically.

The *da_dt* function can then be revised as shown in Figure 19.

The above implementation produces the same output, with the same efficiency, as the previous version that used an explicit if/else statement.

### 2.6. Non-smooth dynamics (advanced): *Aplysia* feeding model

This section presents a final demonstration of the use of pointers with a more advanced example of non-smooth dynamics. The neuromechanical, closed-loop model describes the feeding behavior for the sea hare *Aplysia californica* (Shaw et al., 2015; Lyttle et al., 2017; Wang et al., 2022). The full equations are given in Appendix 1.6 (Supplementary material). The following is a partial list of differential equations for only the state variables that involve the use of pointers.


(5) $$\begin{array}{l} \frac{da_{0}}{dt}=(a_{0}(1-a_{0}-\gamma a_{1})+\mu +\varepsilon_{0}(x_{r}-\xi_{0})\sigma_{0})/\tau_{a}\\ \frac{da_{1}}{dt}=(a_{1}(1-a_{1}-\gamma a_{2})+\mu +\varepsilon_{1}(x_{r}-\xi_{1})\sigma_{1})/\tau_{a}\\ \frac{da_{2}}{dt}=(a_{2}(1-a_{2}-\gamma a_{0})+\mu +\varepsilon_{2}(x_{r}-\xi_{2})\sigma_{2})/\tau_{a}\\ \frac{du_{0}}{dt}=((a_{0}+a_{1})u_{\text{max}}-u_{0})/\tau_{m}\\ \frac{du_{1}}{dt}=(a_{2}u_{\text{max}}-u_{1})/\tau_{m}\\ \frac{dx_{r}}{dt}=(F_{\text{musc}}(u_{0},u_{1},x_{r})+rF_{\text{sw}})/b_{r} \end{array}$$

Here *a*_0_, *a*_1_, and *a*_2_ are neural population firing rates, *u*_0_ and *u*_1_ are muscle activation variables, *x*_*r*_ is the one-dimensional position of a grasping organ, and the following are parameters (see Appendix 1.6 in Supplementary material): γ, μ, ε_*i*_, ξ_*i*_, τ_*a*_, *u*_max_, τ_*m*_, *F*_sw_, and *b*_*r*_. The muscle force *F*_musc_ acting on the grasper is specified in Appendix 1.6 (Supplementary material). We enforce a requirement on the neuronal firing rates *a*_*i*_≥0, *i* = 1, 2, 3, namely that the values are restricted to the range [0, 1]. A similar requirement holds naturally for the muscle activation variables *u*_*i*_, *i* = 1, 2, but does not have to be enforced algorithmically. The grasper state *r* is either open (*r* = 0) or closed (*r* = 1), and switches between these two discrete states when the neural activity crosses a threshold, $a_{1}+a_{2}\geq \frac{1}{2}$. See Appendix 1.6 in Supplementary material for details.

Previous implementations of the model used simulation platforms other than NEURON (Shaw et al., 2015; Lyttle et al., 2017; Wang et al., 2022). In the present NEURON implementation, equations for *a*_0_, *a*_1_, and *a*_2_ are placed in a mechanism named *brain*, and all other equations are placed in a mechanism named *body*. A pointer is necessary for the *brain* mechanism to access the variable *x*_*r*_, which is the physical grasper position. Below is the beginning of the program *brain.mod* that shows the pointer variable *xrPointer* for this purpose.


       :: NMODL code ::      NEURON {        SUFFIX brain        POINTER xrPointer      } 

Pointers are also necessary for the *body* mechanism to access the variables *a*_0_, *a*_1_, and *a*_2_. Below is the beginning of the program *body.mod* that shows the pointer variables *a0Pointer, a1Pointer*, and *a2Pointer* for this purpose.


    :: NMODL code ::   NEURON {     SUFFIX body    POINTER a0Pointer, a1Pointer, a2Pointer   } 

**Figure 20:**
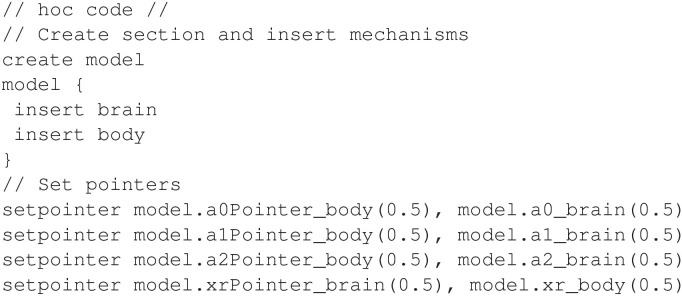
Inserting mechanisms and configuring pointers for the *Aplysia* feeding model.

The mechanisms can be inserted and pointers connected as shown in Figure 20.

**Figure 21:**
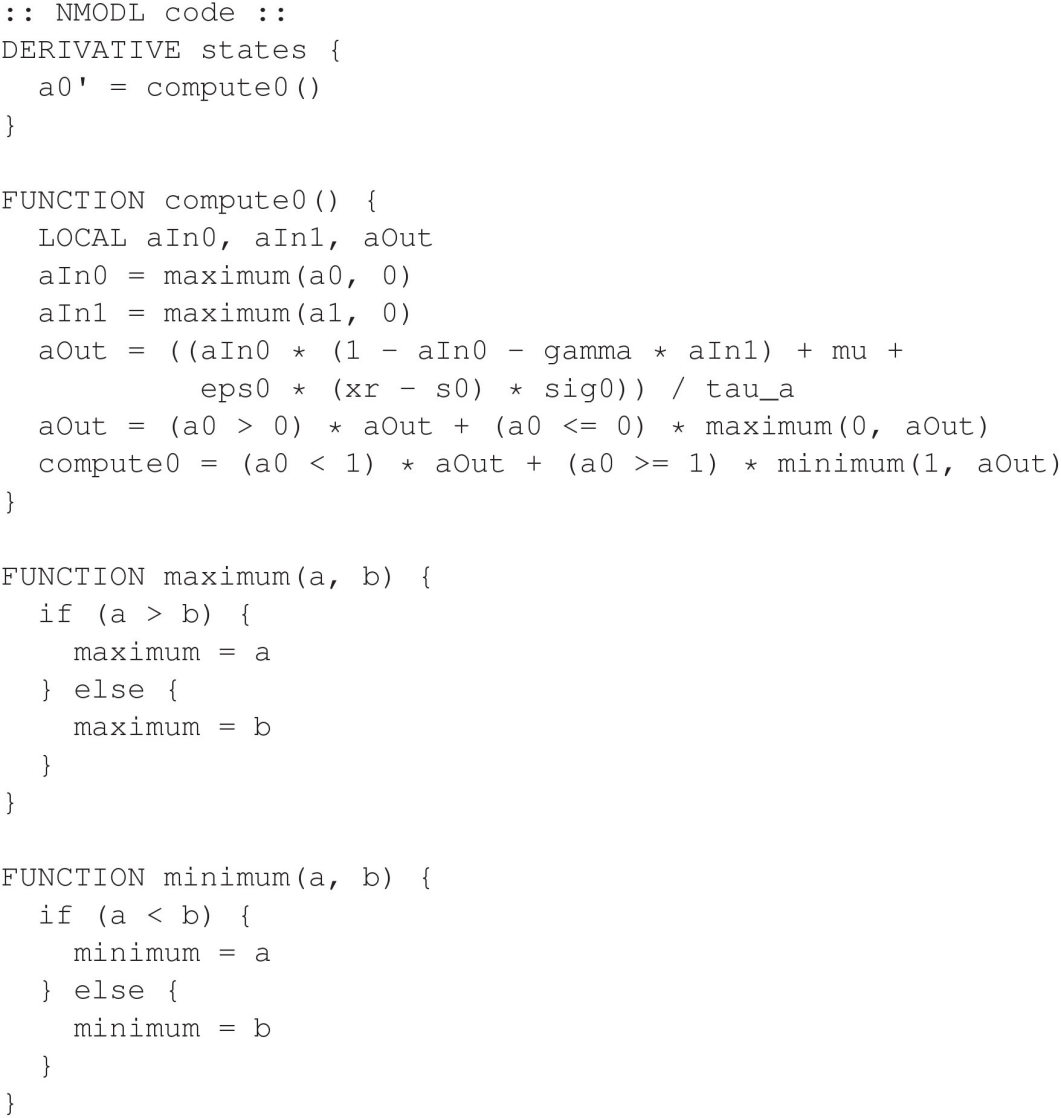
Example from the *brain* mechanism for the *Aplysia* feeding model. This excerpt only includes code for the state variable *a*_0_.

The model requires greater programming complexity than the previously discussed models, due to the nature of the non-smooth dynamics. State variables *a*_0_, *a*_1_, and *a*_2_ require a lower bound of 0, similar to the logic used in the model from Section 2.5. In the present model, there is an additional requirement that these variables have an upper bound of 1. Care is given to also ensure that state variable values used in the differential equations themselves are bounded in the range [0, 1]. NEURON does not include native “min” or “max” functions, so we implement supplementary functions for this purpose within NMODL. Using the Boolean algebra formulation from the end of Section 2.5, Figure 21 shows a partial code listing from the program *brain.mod* that computes the derivative for the state variable *a*_0_.

For state variables *a*_1_ and *a*_2_, the programming is nearly identical except that the right hand side of each differential equation would be different, according to (5). Note that source files are available using the link in Section 4.

When simulating this model, it is useful to consider its multimodal behavior that can be determined by a parameter μ, which appears in Equation (5). For example, μ = 1 × 10^−5^ produces heteroclinic behavior, while μ = 2 × 10^−5^ produces limit cycle behavior (Lyttle et al., 2017). The variable mu is declared as a parameter variable in *brain.mod* with a default value of 1 × 10^−5^. Because it is a parameter variable, its value can be modified at any time without recompiling the NMODL program. For example, the following hoc statement can be used to reconfigure the model for limit cycle behavior using μ = 2 × 10^−5^.


        // hoc code //      mu_brain = 2e-5 

Below is the Python equivalent of this parameter value change.


       # Python code #      h.mu_brain = 2e-5 

**Figure 22:**
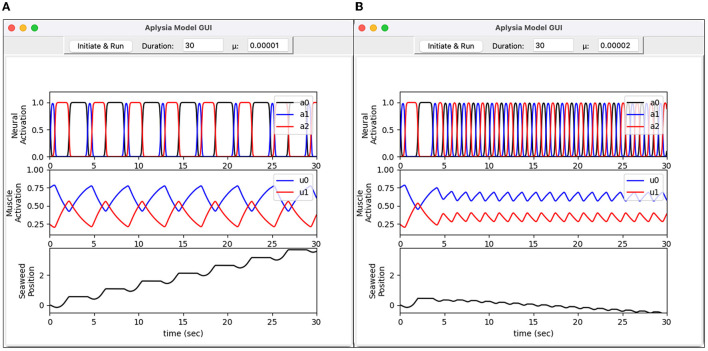
Graphical user interface for *Aplysia* feeding model. Written in Python, the interface allows the user to set simulation parameters and visualize model output. **(A)** Heteroclinic-cycle–like behavior using μ = 0.00001. This activity pattern results in effective intake of seaweed. **(B)** Limit-cycle behavior using μ = 0.00002. This activity pattern is ineffective at drawing in seaweed. Top graphs show activation levels for three neuron pools (*a*_0_, *a*_1_, and *a*_2_). Middle graphs show activation levels for two muscles (*u*_0_ and *u*_1_). Bottom graphs show the simulated seaweed position.

To demonstrate this flexibility, Figure 22 presents a Python graphical user interface (GUI) that allows the user to both view the output and control certain settings for the simulation.

Figure 22 reproduces a key result from Lyttle et al. (2017). For sufficiently small values of the endogenous activation parameter μ, the neural activity (*a*_0_, *a*_1_, *a*_2_) enters a “heteroclinic cycling mode” in which inhibition drives the neural trajectory to collide with the constraint surfaces *a*_*i*_≥0, engaging the non-smooth element of the neural mechanics. The trajectory remains pinned to a constraint surface for an interval dictated by the time it takes for the inhibition—which carries the sensory feedback signal from the grasper to the brain—to release the inhibited neural pool. Under these conditions the system responds robustly to small forces resisting the intake of the seaweed by extending the retraction phase of the motion, as described in Shaw et al. (2015) and Wang et al. (2022). In contrast, when μ is slightly larger, the endogenous activation does not permit inhibition to push the firing rates all the way to zero, so the constraint *a*_*i*_≥0 goes unenforced. Consequently, the progress of the trajectory around its limit cycle orbit is much less sensitive to the effects of an applied force, and the system fails to consume seaweed effectively. Note the downward slope of the seaweed position in Figure 22B, bottom panel, indicating a net *loss* of seaweed.

## 3. Discussion

The models presented here demonstrate two main aspects of our proposed NEURON framework for neuromechanical modeling. First, program organization may be improved in NEURON by separating neural and biomechanical components with the use of pointers. Second, the NEURON environment provides several options for modeling biomechanical mechanisms while offering flexibility in the choice of neuronal models. We demonstrated these concepts using five different models with increasing levels of complexity.

The first two models showcase the general design strategy and programming technique of using pointers. The neuromuscular model in Section 2.1 provides an introduction to pointers in the NMODL language and the complex syntax required to connect two separate NMODL programs. The half-center oscillator system in Section 2.2 is a closed-loop neuromechanical model that demonstrates the capability of connecting pointers to both state variables and parameter variables. The complexity of programming with pointers is further addressed by presenting a Python implementation of these models (see Section 2.3).

The remaining models provide realistic examples of a modular design approach while highlighting important considerations for both neural and biomechanical modeling. We present a neurophysiological, closed-loop model of respiratory control that uses a prominent feature in NEURON for implicit current management (see Section 2.4). In addition to the separation of neural and biomechanical components, this model also demonstrates the use of pointers when using separate NMODL programs for different transmembrane currents. The oscillator model of Section 2.5 serves as an exercise for the reader to understand the basic issues of non-smooth dynamics, which are important in many biomechanical models. Lastly, a neuromechanical, closed-loop model of feeding behavior for *Aplysia californica* (see Section 2.6) demonstrates a realistic, advanced application of non-smooth dynamics. To ensure that the pointers are not affecting stability, the user should always check that halving the step size has little or no effect on results, as discussed above in Section 2.1. We would point out, however, that this would be an appropriate practice to ensure convergence even if one were not using pointers (Brocke et al., 2016).

A limitation to this work is that all biomechanical and physics modeling must be done within the NMODL programming language. There are currently very few biomechanical models available that have been created using NMODL. Additionally, some techniques found in physical modeling platforms are not easily implemented using NMODL. However, such lack of support is a common limitation among neuronal modeling platforms, and physical modeling platforms present a similar limitation in that they will likewise not be practical for neuronal modeling.

Although we have taken the first steps toward showing how NEURON can be integrated with biomechanical modeling, it will ultimately be important to create a unified framework that can both do high quality neuronal network simulations and biomechanics simulations. It is technically possible to do this using NMODL, but other approaches may be more effective and efficient, and should be a focus of future work.

Parameter sensitivity analysis is an important aspect of model building and validation. Several of the models we reproduce from the literature, specifically Diekman et al.'s closed loop respiratory control model and Shaw et al.'s *Aplysia* feeding control model, are taken from papers that did include detailed parameter sensitivity studies. There exists a Python package, Uncertainpy, that is already integrated with NEURON, that is designed specifically for parametric sensitivity analysis (Tennøe et al., 2018).

Despite these limitations, the framework presented here provides a first step toward integrating neural dynamics, as implemented in NEURON, with biomechanics. An important next step would be to interface NEURON with a physics engine in order to facilitate more detailed representations of biomechanical systems. In future work, it will be crucial to establish a convenient framework for specifying neuro-mechanical transfer functions, and also to optimize the choice and parameterization of numerical integrators to handle the combined neural and (possibly non-smooth) biomechanical simulators.

## 4. Methods

The following software versions were used: NEURON 8.2.0 and Python 3.10.5. For the timing analysis in Section 2.5, the processor was a 1.6 GHz Intel Core i5 running Mac OS version 12.6, and the results were consistent across multiple simulation lengths. Code for all of the models is available at https://github.com/fietkiewicz/PointerBuilder.

## Data availability statement

The datasets presented in this study can be found in online repositories. The names of the repository/repositories and accession number(s) can be found below: https://github.com/fietkiewicz/PointerBuilder.

## Author contributions

CF, HC, and PT conceived the research. CF and DC carried out the research. HC and PT supervised the research. CF, RM, HC, and PT wrote the paper. All authors contributed to the article and approved the submitted version.
