# Supplementary material for: Tutorial: using NEURON for neuromechanical simulations
Source: Front Comput Neurosci. 2023 Jul 31;17:1143323. doi: 10.3389/fncom.2023.1143323 (PMC10424731; doi:10.3389/fncom.2023.1143323)
Supplement: Supplementary file 1 [file Presentation_1.pdf]

1 APPENDICES

1.1 Neuromuscular model

Section 2.1 presented a neuromuscular model for the purpose of demonstrating the basic usage of pointers, and section 2.3 used the same model to demonstrate a Python equivalent. Here, we provide additional details about the full model, which is adapted from a NEURON implementation by Kim (Kim and Heckman, accepted; Kim, 2020, 2017) with several modifications.

Kim's muscle model involves interaction between the muscle activation variable *a*, the muscle length variable *xm*, and the timing of the action potentials produced by the presynaptic motor neuron. Kim's implementation achieves modular separation of mechanisms via two innovations: attaching the muscle section directly to the neuron's initial segment, and using ionic currents to represent the biomechanical elements *a* and *xm*. Specifically, Kim's NMODL program for the calcium dynamics accesses the neuron membrane voltage by inserting the calcium mechanism into an additional section of the neuron itself.

The approach of connecting sections is commonly used to allow for differences in properties within a single neuron, such as differences in diameter and ion channels (e.g. dendrite vs. soma vs. axon), but in this case the "muscle" and the "neuron" are not physically connected except via a synaptic connection. In addition, the *force* mechanism depends on the variables in the *calcium* mechanism for activation and position. Using the fact that both mechanisms were inserted into the same section, Kim's implementation cleverly utilized an unrelated feature in NMODL that is designed for modeling ion concentrations in intracellular space. This feature includes specialized WRITE and READ instructions that facilitate the internal management of a collective quantity within the same section.

In contrast, we achieve a more transparent implementation by (i) using the *NetCon* mechanism to communicate spike times from the presynaptic motor neuron to the muscle (which consequently need not be physically connected to the neuron) (Hines and Carnevale, 2004) and (ii) using pointers to allow conceptually distinct biomechanical elements to interact in a natural way.

In addition to the conceptual reorganization of the code, another difference is that the Kim model used a multicompartmental, spatially reconstructed motoneuron with customized ion channel mechanisms, whereas the model presented here uses the *hh* mechanism which is part of the standard NEURON library. Though this difference is not important in the context of using pointers, it highlights the versatility of the NEURON simulation environment. The remainder of this section provides the full model equations and additional code.

1.1.1 Neuromuscular model equations

The equations for calcium dynamics and muscle activation are as in Kim (Kim, 2017). We reproduce the equations below, and provide descriptions of the variables in Table 1. Table 2 provides descriptions of the parameter values.

$$\dot{\mathrm{Ca}}_{\mathrm{SR}} = -K_1 \cdot \mathrm{CS}_0 \cdot \mathrm{Ca}_{\mathrm{SR}} + (K_1 \cdot \mathrm{Ca}_{\mathrm{SR}} + K_2) \cdot \mathrm{Ca}_{\mathrm{SRCS}} - R + U \tag{1}$$

$$\dot{\mathrm{Ca}}_{\mathrm{SRCS}} = K_1 \cdot \mathrm{CS}_0 \cdot \mathrm{Ca}_{\mathrm{SR}} - (K_1 \cdot \mathrm{Ca}_{\mathrm{SR}} + K_2) \cdot \mathrm{Ca}_{\mathrm{SRCS}} \tag{2}$$

$$\dot{\mathrm{Ca}}_{\mathrm{SP}} = -(K_3 \cdot B_0 + K_5 \cdot T_0) \cdot \mathrm{Ca}_{\mathrm{SP}} + (K_3 \cdot \mathrm{Ca}_{\mathrm{SP}} + K_4) \cdot \mathrm{Ca}_{\mathrm{SPB}} + \tag{3}$$

$$(K_5 \cdot \mathrm{Ca}_{\mathrm{SP}} + K_6) \cdot \mathrm{Ca}_{\mathrm{SP}} + R - U \tag{4}$$

$$\dot{\mathrm{Ca}}_{\mathrm{SPB}} = K_3 \cdot B_0 \cdot \mathrm{Ca}_{\mathrm{SP}} - (K_3 \cdot \mathrm{Ca}_{\mathrm{SP}} + K_4) \cdot \mathrm{Ca}_{\mathrm{SPB}} \tag{5}$$

$$\dot{\mathrm{Ca}}_{\mathrm{SPT}} = K_5 \cdot T_0 \cdot \mathrm{Ca}_{\mathrm{SP}} - (K_5 \cdot \mathrm{Ca}_{\mathrm{SP}} + K_6) \cdot \mathrm{Ca}_{\mathrm{SPT}} \tag{6}$$

$$R = \mathrm{Ca}_{\mathrm{SR}} \cdot R_{\mathrm{max}} \cdot \sum_{i=1}^{n} \left(1 - \exp\left(-\frac{t - t_i}{\tau_1}\right)\right) \cdot \exp\left(-\frac{t - t_i}{\tau_2}\right) \tag{7}$$

$$U = U_{\mathrm{max}} \cdot \left(\frac{(\mathrm{Ca}_{\mathrm{SP}})^2 \cdot K^2}{1 + \mathrm{Ca}_{\mathrm{SP}} \cdot K + (\mathrm{Ca}_{\mathrm{SP}})^2 \cdot K^2}\right)^2 \tag{8}$$

$$K_5\left(X_{\mathrm{m}}\right) = \phi\left(X_{\mathrm{m}}\right) \cdot K_{5\mathrm{i}} \tag{9}$$

$$\phi\left(X_{\mathrm{m}}\right) = \begin{cases} \phi_1 \cdot X_{\mathrm{m}} + \phi_2, & \text{for } X_{\mathrm{m}} < -8 \\ \phi_3 \cdot X_{\mathrm{m}} + \phi_4, & \text{for } X_{\mathrm{m}} \geq -8 \end{cases} \tag{10}$$

$$K_6\left(\tilde{A}\right) = \frac{K_{6\mathrm{i}}}{1 + 5 \cdot \tilde{A}} \tag{11}$$

$$\dot{\tilde{A}} = \frac{\tilde{A}_{\infty} - \tilde{A}}{\tau_{\tilde{A}}} \tag{12}$$

$$\tilde{A}_{\infty} = 0.5\left(1 + \tanh\frac{\mathrm{Ca}_{\mathrm{SPT}}/T_0 - C_1}{C_2}\right) \tag{13}$$

$$\tau_{\tilde{A}} = C_3\left(\cosh\frac{\mathrm{Ca}_{\mathrm{SPT}}/T_0 - C_4}{2 \cdot C_5}\right) \tag{14}$$

$$A = \left(\tilde{A}\right)^{\alpha} \tag{15}$$

$$F = P_0 \cdot K_{\mathrm{SE}} \cdot (X_{\mathrm{m}} - X_{\mathrm{CE}}) \tag{16}$$

$$\dot{X}_{\mathrm{CE}} = \begin{cases} \frac{-b_0 \cdot (P_0 \cdot g(X_{\mathrm{m}}) \cdot A - F)}{F + a_0 \cdot g(X_{\mathrm{m}}) \cdot A}, & \text{for } F \leq P_0 \cdot g\left(X_{\mathrm{m}}\right) \cdot A \\ \frac{-d_0 \cdot (P_0 \cdot g(X_{\mathrm{m}}) \cdot A - F)}{2 \cdot P_0 \cdot g(X_{\mathrm{m}}) \cdot A - F + c_0 \cdot g(X_{\mathrm{m}}) \cdot A}, & \text{otherwise} \end{cases} \tag{17}$$

$$g\left(X_{\mathrm{m}}\right) = \exp\left[-\left(\frac{X_{\mathrm{m}} - g_1}{g_2}\right)^2\right] \tag{18}$$

Variable	Initial Value	Description
$\mathrm{Ca_{SR}}$	$0.0025\ M$	calcium in sarcoplasmic reticulum
$\mathrm{Ca_{SRCS}}$	$0\ M$	calcium bound to calsequestrin
$\mathrm{Ca_{SP}}$	$10^{-10}\ M$	calcium in sarcoplasm
$\mathrm{Ca_{SPB}}$	$0\ M$	calcium bound to buffering proteins
$\mathrm{Ca_{SPT}}$	$0\ M$	calcium bound to troponin
R	N/A	calcium release
U	N/A	calcium reuptake
n	N/A	total number of postsynaptic potentials
t_i	N/A	time of ith postsynaptic potential
$\tilde{A}$	N/A	activation level for steady stimulation
A	N/A	activation level for impulse stimulation
F	N/A	muscle force
X_{CE}	N/A	contractile element length

Table 1. Variables for neuromuscular model. Initial values are provided for state variables.

1.1.2 Neuromuscular model program files

Below is the main hoc program that inserts the mechanisms *hh*, *calcium*, and *force*, and connects the pointers, as described in section 2.1. Additionally, the NetStim and ExpSyn classes are used to evoke action potentials in the neuron. Note that the *hh* mechanism is part of the standard NEURON library. For the custom *calcium* and *force* mechanisms, partial details were provided in section 2.1. The complete source files, including Python versions, are available using the link given in Methods, section 4.

```
// Create neuron model
load_file("nrngui.hoc")
cell {
  insert hh
}

// Create stimulus for neuron
objref ns, syn, nc
ns = new NetStim()
ns.interval = 100
syn = new ExpSyn(0.5)
nc = new NetCon(ns, syn)
nc.delay = 0
```

Parameter	Value	Description
X_{m}	$-8\ mm$	total muscle length
K_1	$3000\ M^{-1}m^{-1}$	rate constant for binding Ca^{2+} to calsequestrin
K_2	$3\ ms^{-1}$	rate constant for release of Ca^{2+} from calsequestrin
K_3	$400\ M^{-1}m^{-1}$	rate constant for binding sarcoplasmic Ca^{2+} to buffer proteins
K_4	$1\ ms^{-1}$	rate constant for release of Ca^{2+} from sarcoplasmic buffer proteins
K_5	cf. eqn. (9)	rate constant for binding sacroplasmic Ca^{2+} to troponin
K_6	cf. eqn. (11)	rate constant for release of Ca^{2+} from troponin
$K_{5\mathrm{i}}$	$4 \cdot 10^{-5}\ M^{-1}m^{-1}$	initial value of rate constant K_5
$K_{6\mathrm{i}}$	$150\ ms^{-1}$	initial value of rate constant K_6
CS_0	$0.03\ M$	total concentration of calsequestrin
B_0	$0.00043\ M$	total concentration of buffering protein
T_0	$0.00007\ M$	total concentration of troponin
K	$850\ M^{-1}$	site binding constant for pump activation
R_{max}	$10\ ms^{-1}$	peak release
U_{max}	$10\ M^{-1}m^{-1}$	peak reuptake
ϕ_1	$0.03\ mm^{-1}$	K5 scaling, less than optimal length
ϕ_2	1.23	K5 offset, less than optimal length
ϕ_3	$0.01\ mm^{-1}$	K5 scaling, optimal length or greater
ϕ_4	1.08	K5 offset, optimal length or greater
C_1	0.128	normalized Ca_{SPT} for half muscle activation
C_2	0.093	slope of the activation curve at C_1
C_3	61.206	scaling factor for temperature,
C_4	-13.116	normalized Ca_{SPT} for maximum time constant
C_5	5.095	width scaling factor for $\tau_{\tilde{A}}$
α	2	exponent for likelihood of cross-bridge formation
P_0	$23\ N$	peak force at optimal length
K_{SE}	0.4	muscle stiffness parameter
a_0	$2.35\ N$	Hill-Mashima equation (17) coefficient
b_0	$24.35\ mm \cdot s^{-1}$	Hill-Mashima equation (17) coefficient
c_0	$-7.4\ N$	Hill-Mashima equation (17) coefficient
d_0	$30.3\ mm \cdot s^{-1}$	Hill-Mashima equation (17) coefficient
g_1	$-8\ mm$	length-tension curve offset coefficient
g_2	$21.4\ mm$	length-tension curve scaling coefficient

Table 2. Parameter values for neuromuscular model.

```
    nc.weight = 2

    // Create muscle model
    objref calciumObject, forceObject
```

```
    create muscle
    muscle {
        calciumObject = new calcium(0.5)
        forceObject = new force(0.5)
    }

    // connect neuron to muscle
    objref neuromuscularJunction
    neuromuscularJunction = new NetCon(&cell.v(0.5), calciumObject)
    neuromuscularJunction.threshold = -40

    // Set pointers
    setpointer forceObject.aPointer, calciumObject.A
    setpointer forceObject.xmPointer, calciumObject.xm
```

1.2 Converting a single mod file

It is common to begin with an existing mod file that has a system of equations but does not use pointers. Here we present a particularly simple example of converting a single mod file into multiple files through the use of pointers. The model consists of the two-dimensional, nonlinear system of the Lotka-Volterra equations, also known as the predator-prey equations, given below:

$$\begin{aligned} \frac{da}{dt} &= \alpha\, a - \beta\, ab \\ \frac{db}{dt} &= \gamma\, ab - \delta\, b \end{aligned} \tag{19}$$

where a is interpreted as a prey species, and b is interpreted as a predator species. For concreteness, we will use parameter values $\alpha = 1.1$ (prey intrinsic birth rate), $\beta = 0.4$ (predation effect on prey), $\gamma = 0.1$ (predation benefit to predator), $\delta = 0.4$ (predator intrinsic death rate).

Using a single file, the model above might be implemented as shown below, where the mechanism is named *model*.

```
    :: NMODL code ::

    NEURON {
     SUFFIX model :: Custom name for mechanism
    }

    STATE { a b } :: Declare state variables.

    INITIAL {  :: Set initial values.
     a = 10.0
     b = 10.0
    }

    BREAKPOINT {
```

```
  SOLVE states METHOD derivimplicit
 }

DERIVATIVE states {
  a' = 1.1 * a - 0.4 * a * b
  b' = 0.1 * a * b - 0.4 * b
 }
```

Next, we place each differential equation in a separate file using a POINTER statement in the NEURON block and placing the name of the pointer variable in the ASSIGNED block. Figure 1 shows the implementation using separate mechanisms named *prey* and *predator*.

```
NEURON {
   SUFFIX prey
   POINTER bPointer
}

PARAMETER {}

ASSIGNED { bPointer }

STATE { a }

BREAKPOINT {
   SOLVE states METHOD derivimplicit
}

INITIAL { a = 10 }

DERIVATIVE states {
   a' = 1.1 * a - 0.4 * a * bPointer
}
```

```
NEURON {
   SUFFIX predator
   POINTER aPointer
}

PARAMETER {}

ASSIGNED { aPpointer }

STATE { b }

BREAKPOINT {
   SOLVE states METHOD derivimplicit
}

INITIAL { b = 10 }

DERIVATIVE states {
   b' = 0.1 * aPointer * b - 0.4 * b
}
```

Figure 1. NMODL programs for Lotka-Volterra equations, demonstrating pointers. (a) Program prey.mod. (b) Program predator.mod.

The following hoc instructions create a section, insert the *prey* and *predator* mechanisms, and connect the pointers:

```
// hoc code //
create model
model {
 insert prey
 insert predator
}
setpointer model.aPointer_predator(0.5), model.a_prey(0.5)
setpointer model.bPointer_prey(0.5), model.b_predator(0.5)
```

A graph of the results is shown in Figure 2. Note that source files are available using the link in Methods, section 4.

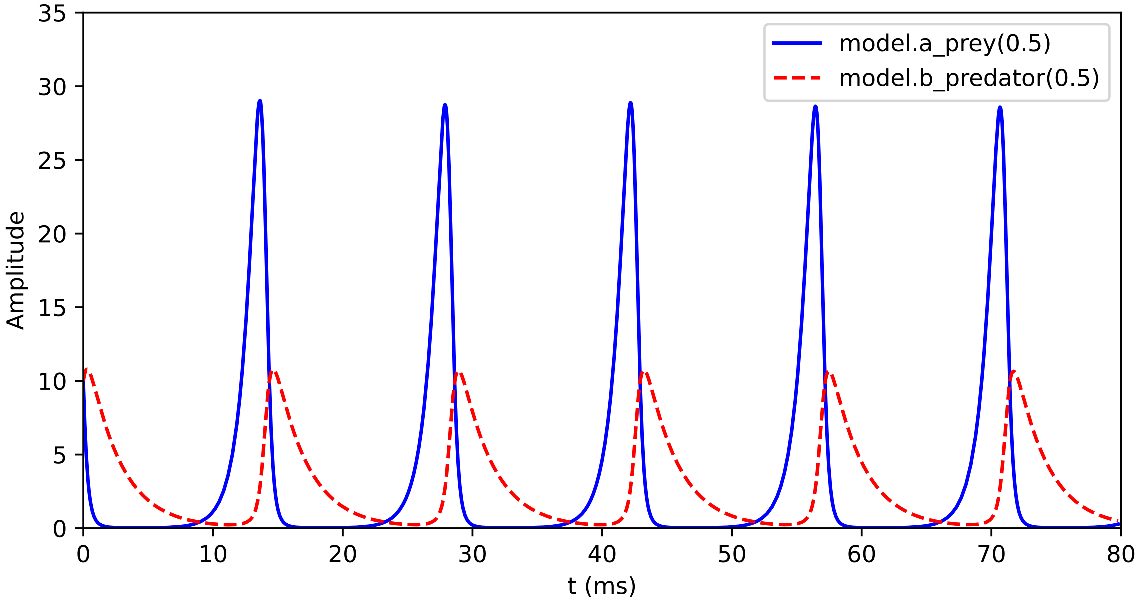

Figure 2. Simulation of Lotka-Volterra equations using the mechanisms prey and predator, as defined in Figure 1. The legend shows the exact hoc syntax used to access the state variable a in prey.mod (solid line) and b in predator.mod (dashed line). The simulation used the variable time step option in NEURON with the absolute tolerance set to 0.0001. In this example, the position parameter (0.5) can be any number between zero and one.

1.3 PointerBuilder application

NEURON provides graphical user interfaces (GUIs) that users may elect to use for creating and simulating models. However, there currently are no such tools for working with pointers. Here we present tools to facilitate the writing of setpointer instructions. The complete source files are available using the link given in Methods, section 4.

Writing a setpointer instruction has two particular complications. First, it requires the names of multiple variables, mechanisms, and sections, for a total of six separate pieces of information. Second, the syntax for combining these pieces takes multiple forms, depending on the type of variable (state or parameter) and programming language (hoc or Python). We present two GUI applications, for hoc and Python respectively, that can help a modeler organize the required components and automatically generate the correct syntax. Source code for the tools is provided, and interested users can customize them if desired.

The first tool is intended for users who wish to work with the hoc language. It is written using the native NEURON library for GUI development and does not require Python. The interface is shown in Figure 3, using the predator-prey model from sections 2.1 and 2.2 as an example. The six required components are grouped in a horizontal format that resembles the syntax of an actual setpointer instruction. Separate rows distinguish between the pointer and either a state or parameter variable, and columns indicate the three components required for each variable: NMODL file, variable name, and section. The user can complete the fields manually, or they may use an automatic file scan by clicking a button labeled “Read .mod file”. When performing an automatic file scan, the user first chooses an NMODL file. After selecting a file, a pop up window allows the user to choose from a list of available variables found in the NMODL program. Buttons in the bottom row of the main GUI are used to either save or display the resulting hoc instruction (see Figure 4). The option to “Make .hoc file” saves the hoc instruction to a file which can be automatically

loaded by other hoc programs. The option to "View .hoc command" displays the hoc instruction in the window and in the NEURON terminal window. Note that NEURON allows direct text copying from the terminal window but not from the GUI.

The second tool is intended for users who wish to work with the Python language. It is written using the native GUI tool for Python, called Tkinter, and is shown in Figure 5. The layout and action buttons are similar to those in the hoc tool (see Figure 3), except that a Python command is generated instead. When viewing the Python command, as seen in Figure 6, the user can copy text directly from the window. The Python command can also be saved to a Python file that can be imported by other Python programs.

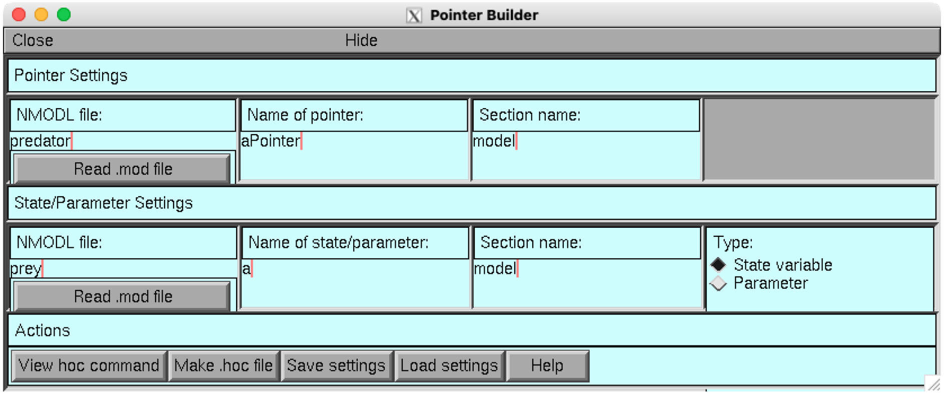

Figure 3. Pointer Builder (NEURON version) is a tool for creating *setpointer* instructions in NEURON. The example shown is based on the NMODL programs in Figure 1 for the Lotka-Volterra model. The interface is comprised of three groups of controls: Pointer Settings, State/Parameter Settings, and Actions. The "Pointer Settings" indicate that the *predator* mechanism contains the pointer variable *aPointer*, and is inserted in the section *model*. The "State/Parameter Settings" indicate that the *prey* mechanism contains the state variable *a*, and is inserted in the section *model*. Available "Actions" include viewing the hoc command, saving the command as a hoc file, saving and loading Pointer Builder settings, and reading help documentation.

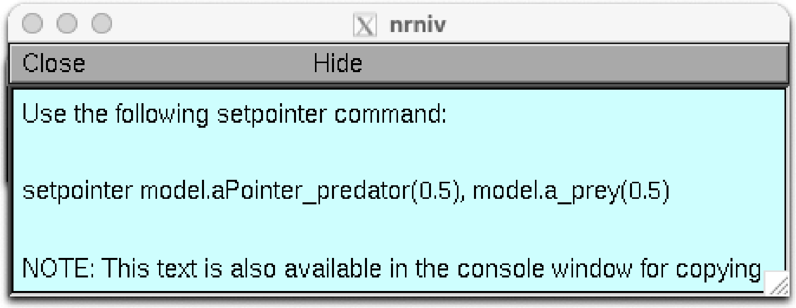

Figure 4. Pointer Builder output for hoc command. A complete setpointer command is displayed after selecting the option to "View hoc command". The text can also be copied from a separate console window (not shown).

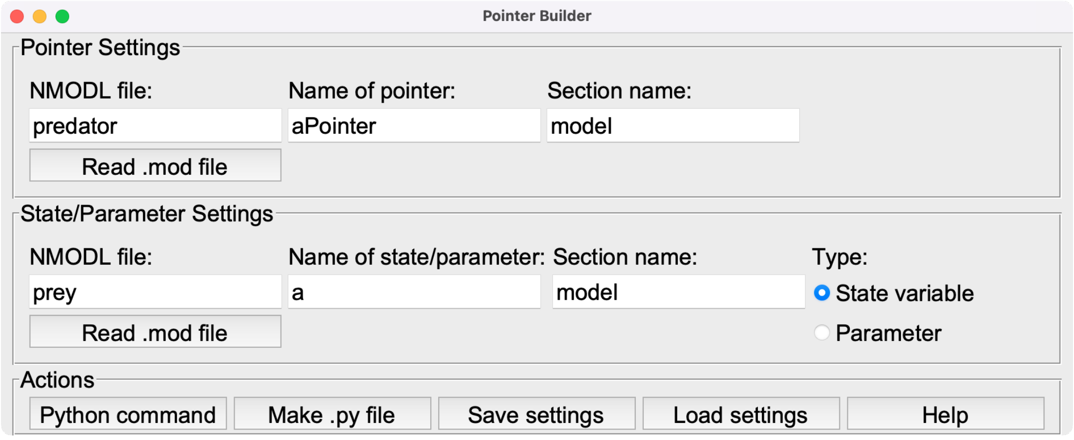

Figure 5. Pointer Builder (Python version) is a tool for configuring pointers in Python. The example shown is based on the NMODL programs in Figure 1 for the Lotka-Volterra model. The interface is comprised of three groups of controls: Pointer Settings, State/Parameter Settings, and Actions. The "Pointer Settings" indicate that the *predator* mechanism contains the pointer variable *aPointer*, and is inserted in the section *model*. The "State/Parameter Settings" indicate that the *prey* mechanism contains the state variable *a*, and is inserted in the section *model*. Available "Actions" include viewing the Python command, saving the command as a Python file, saving and loading Pointer Builder settings, and reading help documentation.

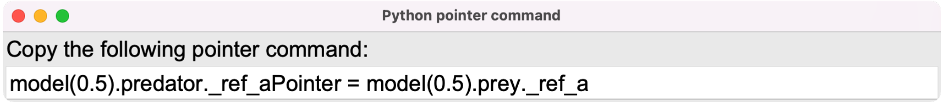

Figure 6. Python Pointer Builder output. A complete command for setting the pointer is displayed after selecting the "Python command" option. The text can be copied directly from the window.

1.4 Half-Center Oscillator Model Description and Program Files

Section 2.2 presented a neuromechanical, closed-loop model of a half-center oscillator coupled to a rudimentary motor system. For the custom *brain* and *body* mechanisms, partial details were provided in section 2.2, and the complete source files are available using the link given in Methods, section 4. For the full model specification, see (Yu and Thomas, 2021). We reproduce the basic equations here. The model comprises two Morris-Lecar type models each with membrane potential V_i and potassium gating variable N_i, $i \in \{1, 2\}$. For cell $i \in \{1, 2\}$ $(i \neq j)$,

$$C\frac{dV_i}{dt} = I_{\text{ext}} - g_{\text{L}}(V_i - E_{\text{L}}) - g_{\text{Ca}}M_\infty(V_i)(V_i - E_{\text{Ca}}) - g_{\text{K}}N_i(V_i - E_{\text{K}})$$
$$- g_{\text{syn}}^{\text{CPG}} S_\infty^{\text{CPG}}(V_j)\left(V_i - E_{\text{syn}}^{\text{CPG}}\right) - g_{\text{syn}}^{\text{FB}} S_\infty^{\text{FB}}(L_j)(V_i - E_{\text{syn}}^{\text{FB}}), \tag{20}$$

$$\frac{dN_i}{dt} = \lambda_N(V_i)(N_\infty(V_i) - N_i), \tag{21}$$

where C is the capacitance, I_{ext} is an applied current, g_{L} is the passive leak conductance, g_{Ca} is the maximal calcium conductance, M_∞ is the voltage-dependent fraction of open calcium channels, E_{X}, $\text{X} \in \{\text{Ca}, \text{K}, \text{L}\}$ is the reversal potential for each current, respectively, and g_{K} is the maximal potassium conductance. The coupling function $S_\infty^{\text{CPG}}(V_j)$ is a saturating sigmoidal function that specifies the impact of the voltage of cell j on the membrane dynamics of cell i. The strength of the inhibitory synapse forming the HCO is controlled by $g_{\text{syn}}^{\text{CPG}}$, and the sensory feedback from the "body" to the "brain" is given by $g_{\text{syn}}^{\text{FB}} S_\infty^{\text{FB}}(L_j)(V_i - E_{\text{syn}}^{\text{FB}})$. In

addition,

$$M_\infty(V_i) = \frac{1}{2}\left(1 + \tanh\left(\frac{V_i - E_1}{E_2}\right)\right), \tag{22}$$

$$N_\infty(V_i) = \frac{1}{2}\left(1 + \tanh\left(\frac{V_i - E_3}{E_4}\right)\right), \tag{23}$$

$$\lambda_N(V_i) = \phi_N\left(\cosh\left(\frac{V_i - E_3}{2E_4}\right)\right), \tag{24}$$

$$S_\infty^{\text{CPG}}(V_j) = \frac{1}{2}\left(1 + \tanh\left(\frac{V_j - E_{\text{thresh}}}{E_{\text{slope}}}\right)\right), \tag{25}$$

$$S_\infty^{\text{FB}}(L_j) = 1 - \frac{1}{2}\tanh\left(\frac{L_j - x_0\ell}{L_{\text{slope}}}\right). \tag{26}$$

The force generated by muscle i is

$$F_i(t) = F_0 \cdot a_i(t) \cdot LT(L_i(t)). \tag{27}$$

Each mucle has a length-tension curve given by

$$LT(L_i(t)) = -\left(5.27 \times 10^{-4}\right)\left(L_i(t)/\ell\right)^2 + 0.1054(L_i(t)/\ell) - 4.27, \tag{28}$$

$$L_i(t) = (50 \pm 0.8x(t))\ell, \quad L_j(t) = (50 \mp 0.8x(t)\ell, \quad -50 < x < 50, \tag{29}$$

where ℓ is a fixed, nominal length value, and $-50 < x < 50$ is the position of the "limb" driven by the opposing muscle forces. Muscle activation a_i is described by the variable

$$a_i(t) = g[A_i(t) - a_0], \qquad 0 < a_i \leq 1, \tag{30}$$

where

$$dA_i/dt = \tau^{-1}\{U_i - [\beta + (1 - \beta)U_i]A_i\}, \tag{31}$$

$$U_i = 1.03 - 4.31e^{(-0.198u_i)}, \qquad 0 \leq U_i \leq 1, \tag{32}$$

$$u_i = (1/2)V_i. \tag{33}$$

Finally, the position of the "limb" is determined by the balance of forces:

$$\frac{dx}{dt} = \frac{1}{b\ell}(F_2 - F_1). \tag{34}$$

1.5 Closed-loop respiratory control model

Section 2.4 presented a closed-loop model of neural control for respiration. Here we provide the equations for the model introduced in Diekman et al. (2017). For the custom mechanisms, partial details were provided in section 2.4, and the complete source files are available using the link given in Methods, section 4.

Central Pattern Generator (CPG): The model in Diekman et al. (2017) used the original Butera-Rinzel-Smith (BRS) model (referred to as "model 1" in Butera et al. (1999)) as a nominal representation of bursting pacemaker neurons in the pre-Bötzinger complex. The CPG comprises a membrane potential V, and dynamical gating variables n (a delayed rectifier potassium (I_{K}) channel activation) and h (persistent sodium (I_{NaP}) channel inactivation). The model sets the "instantaneous" gating variables p_∞ (I_{NaP} activation) and m_∞ (fast sodium (I_{Na}) activation) to be equal to their voltage-dependent asymptotic values. The I_{Na} inactivation gate is set equal to $(1-n)$. The model also includes a leak current (I_{L}) and a tonic excitatory (I_{tonic}) current. All together, we have

$$C\frac{dV}{dt} = -I_{\text{K}} - I_{\text{NaP}} - I_{\text{Na}} - I_{\text{L}} - I_{\text{tonic}} \tag{35}$$

$$\frac{dn}{dt} = \frac{n_\infty(V) - n}{\tau_{\text{n}}(V)} \tag{36}$$

$$\frac{dh}{dt} = \frac{h_\infty(V) - h}{\tau_{\text{h}}(V)} \tag{37}$$

$$I_{\text{K}} = g_{\text{K}} n^4 (V - E_{\text{K}}) \tag{38}$$

$$I_{\text{NaP}} = g_{\text{NaP}} p_\infty(V) h (V - E_{\text{Na}}) \tag{39}$$

$$I_{\text{Na}} = g_{\text{Na}} m_\infty^3(V)(1-n)(V - E_{\text{Na}}) \tag{40}$$

$$I_{\text{L}} = g_{\text{L}}(V - E_{\text{L}}) \tag{41}$$

$$I_{\text{tonic}} = g_{\text{tonic}}(V - E_{\text{tonic}}) \tag{42}$$

$$x_\infty(V) = \frac{1}{1 + \exp[(V - \theta_{\text{x}})/\sigma_{\text{x}}]} \tag{43}$$

$$\tau_{\text{x}} = \frac{\bar{\tau}_{\text{x}}}{\cosh[(V - \theta_{\text{x}})/2\sigma_{\text{x}}]} \tag{44}$$

with parameters $C = 21$ pF, $g_{\text{K}} = 11.2$ nS, $g_{\text{NaP}} = 2.8$ nS, $g_{\text{Na}} = 28$ nS, $g_{\text{L}} = 2.8$ nS, $E_{\text{K}} = -85$ mV, $E_{\text{Na}} = 50$ mV, $E_{\text{L}} = -65$ mV, $E_{\text{tonic}} = 0$ mV, $\theta_{\text{n}} = -29$ mV, $\sigma_{\text{n}} = -4$ mV, $\theta_{\text{p}} = -40$ mV, $\sigma_{\text{p}} = -6$ mV, $\theta_{\text{h}} = -48$ mV, $\sigma_{\text{h}} = 6$ mV, $\theta_{\text{m}} = -34$ mV, $\sigma_{\text{m}} = -5$ mV, $\bar{\tau}_{\text{n}} = 10$ ms, and $\bar{\tau}_{\text{h}} = 10,000$ ms.

Motor pool activity: The BRS cell's membrane potential (V) provides the output of the CPG, driving the respiratory muscles through synaptic activation of a motor unit (α):

$$\frac{d\alpha}{dt} = r_{\text{a}}[T](1-\alpha) - r_{\text{d}}\alpha \tag{45}$$

$$[T] = \frac{T_{\max}}{(1 + \exp(-(V - V_{\text{T}})/K_{\text{p}}))}. \tag{46}$$

Here, we set $r_\mathrm{a} = r_\mathrm{d} = 0.001$ mM^{-1} ms^{-1} giving the rise and decay rate of the synaptic conductance. Variable $[T]$ represents the neurotransmitter concentration, with parameters $T_\mathrm{max} = 1$ mM, $V_\mathrm{T} = 2$ mV, and $K_\mathrm{p} = 5$ mV (Ermentrout and Terman, 2010).

Lung volume: The output of the motor unit determines the rise and fall of lung volume ($\mathrm{vol_L}$):

$$\frac{d}{dt}(\mathrm{vol_L}) = E_1\alpha - E_2(\mathrm{vol_L} - \mathrm{vol_0}). \tag{47}$$

Here $\mathrm{vol_0} = 2$ L is the volume of the unloaded lung, and parameters $E_1 = 0.4$ L and $E_2 = 0.0025$ ms^{-1} were chosen so that the lung expansion would remain in a physiologically reasonable range (West, 2008).

Lung oxygen: At standard atmospheric pressure (760 mmHg), external air with 21% oxygen content registers a partial pressure of oxygen of $P_\mathrm{ext}\mathrm{O_2} = 149.7$ mmHg. As the lungs expand $\left(\frac{d}{dt}[\mathrm{vol_L}] > 0\right)$, they draw in external air. The model makes the simplifying assumption that this fresh air mixes instantaneously with the air already present in the lungs. Therefore, the partial pressure of oxygen in the lung alveoli ($P_\mathrm{A}\mathrm{O_2}$) increases at a rate given by the pressure difference between external and internal air, and by the lung volume. During exhalation, on the other hand, $\left(\frac{d}{dt}[\mathrm{vol_L}] \leq 0\right)$, no external air enters the lungs, so the mixing of air stops. During both contraction and expansion of the lung, oxygen moves between the lungs and the blood. The flux of oxygen from the lungs to the blood occurs at a rate determined by the time constant $\tau_{LB} = 500$ ms, and by the difference in partial pressure of $\mathrm{O_2}$ between the lungs ($P_\mathrm{A}\mathrm{O_2}$) and the arterial blood ($P_\mathrm{a}\mathrm{O_2}$). The rate of change in $P_\mathrm{a}\mathrm{O_2}$ is given by:

$$\frac{d}{dt}(P_\mathrm{A}\mathrm{O_2}) = \frac{P_\mathrm{ext}\mathrm{O_2} - P_\mathrm{A}\mathrm{O_2}}{\mathrm{vol_L}}\left[\frac{d}{dt}(\mathrm{vol_L})\right]_+ - \frac{P_\mathrm{A}\mathrm{O_2} - P_\mathrm{a}\mathrm{O_2}}{\tau_\mathrm{LB}} \tag{48}$$

where the notation $[x]_+$ indicates $\max(x, 0)$.

Blood oxygen: To represent the change in $P_\mathrm{a}\mathrm{O_2}$, we have

$$\frac{d}{dt}(P_\mathrm{a}\mathrm{O_2}) = \frac{J_\mathrm{LB} - J_\mathrm{BT}}{\zeta\left(\beta_{\mathrm{O_2}} + \eta\frac{\partial \mathrm{SaO_2}}{\partial P_\mathrm{a}\mathrm{O_2}}\right)}. \tag{49}$$

(The fluxes of oxygen from the blood to the tissues (J_LB) and from the lungs to the blood (J_LB) have units of moles of $\mathrm{O_2}$ per millisecond.) The denominator converts changes in the number of moles of $\mathrm{O_2}$ in the blood to changes in $P_\mathrm{a}\mathrm{O_2}$. To calculate the flux J_LB, the model assumes the ideal gas law $PV = nRT$, where n is the number of moles of $\mathrm{O_2}$, $R = 62.364$ L mmHg K^{-1} mol^{-1} is the universal gas constant, and $T = 310$ K is temperature. The resulting flux depends on the difference in oxygen partial pressure between the lungs and the blood:

$$J_\mathrm{LB} = \left(\frac{P_\mathrm{A}\mathrm{O_2} - P_\mathrm{a}\mathrm{O_2}}{\tau_\mathrm{LB}}\right)\left(\frac{\mathrm{vol_L}}{RT}\right). \tag{50}$$

Note that the term J_BT accounts for both dissolved and hemoglobin-bound oxygen in the blood:

$$J_\mathrm{BT} = M\zeta\left(\beta_{\mathrm{O_2}}\, P_\mathrm{a}\mathrm{O_2} + \eta\, \mathrm{SaO_2}\right). \tag{51}$$

Following Henry's law, the model takes the concentration of dissolved oxygen in the blood to be directly proportional to $P_\mathrm{a}\mathrm{O}_2$. The blood solubility coefficient, $\beta_{\mathrm{O}_2} = 0.03$ ml O_2 $\times$ L blood^{-1} mmHg^{-1} for blood at 37 degrees C, is the constant of proportionality. Most of the blood's stored oxygen is bound to hemoglobin (Hb). Cooperative binding of oxygen to the four binding sites in each hemoglobin molecule leads to a sigmoidal hemoglobin saturation curve SaO_2:

$$\mathrm{SaO}_2 = \frac{P_\mathrm{a}\mathrm{O}_2^c}{P_\mathrm{a}\mathrm{O}_2^c + K^c} \tag{52}$$

$$\frac{\partial \mathrm{SaO}_2}{\partial P_\mathrm{a}\mathrm{O}_2} = c P_\mathrm{a}\mathrm{O}_2^{c-1} \left(\frac{1}{P_\mathrm{a}\mathrm{O}_2^c + K^c} - \frac{P_\mathrm{a}\mathrm{O}_2^c}{(P_\mathrm{a}\mathrm{O}_2^c + K^c)^2} \right). \tag{53}$$

The phenomenological parameters $K = 26$ mmHg and $c = 2.5$ are taken from Keener and Sneyd (2009).

The model includes a parameter M in (51) to capture the rate of metabolic demand for oxygen from the tissues, in units of ms^{-1}. Equations (49) and (51) include conversion factors ζ and η that depend on the concentration of hemoglobin, $[\mathrm{Hb}] = 150$ gm L^{-1}, as well as the volume of blood, $\mathrm{vol}_\mathrm{B} = 5$ L, respectively. The model assumes a molar oxygen volume of 22.4 L. It is assumed that each fully saturated hemoglobin molecule carries 1.36 ml of O_2 per gram:

$$\zeta = \mathrm{vol}_\mathrm{B} \times \left(\frac{\text{mole } \mathrm{O}_2}{22,400 \text{ mL } \mathrm{O}_2} \right) \tag{54}$$

$$\eta = [\mathrm{Hb}] \times \left(\frac{1.36 \text{ mL } \mathrm{O}_2}{\text{gm Hb}} \right). \tag{55}$$

Chemosensation: Chemosensory feedback from peripheral chemoreceptors in the carotid bodies, carried to brainstem respiratory circuits via the carotid sinus nerve, completes the closed-loop model. These receptors detect reductions in $P_\mathrm{a}\mathrm{O}_2$ and drive the central rhythm generator, as described in more detail in Diekman et al. (2017). The model takes the nonlinear relationship between carotid chemosensory nerve fiber activity and $P_\mathrm{a}\mathrm{O}_2$ to be a sigmoidal saturating function, with the firing rate low until $P_\mathrm{a}\mathrm{O}_2$ is reduced below a threshold (normally about 100 mm Hg) and then steep firing rate increases as $P_\mathrm{a}\mathrm{O}_2$ is reduced further (Hlastala and Berger, 2001; West, 2008). The model captures this behavior via a sigmoidal function connecting $P_\mathrm{a}\mathrm{O}_2$ with the conductance representing external drive to the CPG (g_tonic):

$$g_\text{tonic} = \phi \left(1 - \tanh \left(\frac{P_\mathrm{a}\mathrm{O}_2 - \theta_g}{\sigma_g} \right) \right). \tag{56}$$

Here, $\phi = 0.3$ nS, $\theta_g = 85$ mmHg, and $\sigma_g = 30$ mmHg. This conductance closes the control loop, since $I_\text{tonic} = g_\text{tonic}(V - E_\text{tonic})$ is a term in the CPG voltage equation (35).

1.6 SLG model for *Aplysia* feeding

Section 2.6 presented a closed-loop model of feeding behavior in the sea hare *Aplysia californica* that incorporates biologically-motivated nonsmooth dynamics. For the full model specification, see (Shaw et al., 2015; Lyttle et al., 2017; Wang et al., 2022). We reproduce the basic equations here. For the custom

brain and *body* mechanisms, partial details were provided in section 2.6, and the complete source files are available using the link given in Methods, section 4.

The dynamics of the nominal *Aplysia* feeding model, also known as the SLG model (Shaw et al., 2015; Lyttle et al., 2017; Wang et al., 2022), is given by the following differential equations:

$$
\begin{aligned}
\frac{da_0}{dt} &= (a_0(1 - a_0 - \gamma a_1) + \mu + \varepsilon_0(x_r - \xi_0)\sigma_0)/\tau_a \\
\frac{da_1}{dt} &= (a_1(1 - a_1 - \gamma a_2) + \mu + \varepsilon_1(x_r - \xi_1)\sigma_1)/\tau_a \\
\frac{da_2}{dt} &= (a_2(1 - a_2 - \gamma a_0) + \mu + \varepsilon_2(x_r - \xi_2)\sigma_2)/\tau_a \\
\frac{du_0}{dt} &= ((a_0 + a_1)u_{\max} - u_0)/\tau_m \\
\frac{du_1}{dt} &= (a_2 u_{\max} - u_1)/\tau_m \\
\frac{dx_r}{dt} &= (F_{\text{musc}}(u_0, u_1, x_r) + rF_{\text{sw}})/b_r \\
\frac{dx_{sw}}{dt} &= \begin{cases} -(F_{\text{musc}}(u_0, u_1, x_r) + rF_{\text{sw}})/b_r, a_1 + a_2 \geq 0.5 \\ \hfill 0, a_1 + a_2 < 0.5 \end{cases}
\end{aligned} \tag{57}
$$

The variables a_i represent the firing rates of three nominal populations of motor neurons, active during the "protraction-open" phase (a_0), the "protraction-closed" phase (a_1), and the "retraction" phase (a_2). We supplement the differential equations (57) with hard or "sliding" boundary conditions $a_i \geq 0$, to account for the fact that inhibition cannot drive a firing rate to negative values. When the firing rate of the ith neural pool, a_i, is equal to zero, further inhibition (which would lead to $da_i/dt < 0$ in (57)) is censored, but when excitation overcomes inhibition ($da_i/dt > 0$) the restriction clamping $a_i \equiv 0$ is released. The model includes a simplified version of the feeding apparatus biomechanics. The animal pulls in food using the radula/odontophore, or "grasper". In the model, the grasper can either be open ($r = 0$) or closed ($r = 1$). This Boolean switching term appears in the differential equation for the grasper position, dx_r/dt. The protraction ($dx_r/dt > 0$) or retraction ($dx_r/dt < 0$) of the grasper is determined by the balance of forces produced by the protractor muscle activation (u_0) and retractor muscle activation (u_1). The net force exerted by the muscles is given by the sum of the two muscle forces

$$
\begin{aligned}
F_{\text{musc}}(u_0, u_1, x_r) &= F_{\text{musc,pro}} + F_{\text{musc,ret}} \\
&= k_0 \phi\left(\frac{c_0 - x_r}{w_0}\right) u_0 + k_1 \phi\left(\frac{c_1 - x_r}{w_1}\right) u_1
\end{aligned} \tag{58}
$$

where

$$
\phi(x) = -\frac{3\sqrt{3}}{2} x(x-1)(x+1)
$$

is the effective length-tension curve for muscle forces, c_i, w_i and k_i denote the mechanical properties of each muscle.

The seaweed is subject to an external force resisting ingestion, F_{sw}. This force only affects the grasper when the grasper is closed ($r = 1$). This condition is set by a threshold, ($r = 1 \iff a_1 + a_2 > 0.5$).

When the grasper is open ($a_1 + a_2 \leq 0.5$), $r = 0$, and the grasper moves independently of the seaweed. Values for model parameters and initial conditions are given in Table 3 and Table 4 below, repsectively. For additional details on the biological assumptions motivating the model, see Shaw et al. (2015); Lyttle et al. (2017); Wang et al. (2022).

1.7 Tables

Parameter	Value	Description
γ	2.4	inhibition strength from next pool
ϵ_i	10^{-4}	sensory feedback strength
μ	10^{-6}	neural pool intrinsic excitation
τ_a	0.05	neural pool time constant
τ_m	2.45	muscle activation time constant
b_{r}	0.4	grasper damping constant
c_0	1.0	position of shortest length for I2
c_1	1.1	position of center of I3
F_{sw}	0.01	force on the seaweed resisting ingestion
σ_0	-1	sign of proproceptive input to a_0 motor pool
σ_1	1	sign of proproceptive input to a_1 motor pool
σ_2	1	sign of proproceptive input to a_2 motor pool
ξ_0	0.5	proprioceptive neutral position for protraction-open neural pool
ξ_1	0.5	proprioceptive neutral position for protraction-closed neural pool
ξ_2	0.25	proprioceptive neutral position for retraction-closed neural pool
u_{max}	1.0	maximum muscle activation
w_0	2	maximal effective length of I2
w_1	1.1	maximal effective length of I3
k_0	1	strength and direction of the protrator muscle
k_1	-1	strength and direction of the retractor muscle

Table 3. *Aplysia* feeding model parameters, reproduced from (Wang et al., 2022).

State variable	Initial value	Description
a_0	0.9	activity of I2 motor pool (non-negative)
a_1	0.08355	activity of hinge motor pool (non-negative)
a_2	0.00003	activity of I3 motor pool (non-negative)
u_0	0.748	activity of I2 muscle
u_1	0.25	activity of I3 muscle
x_r	0.65	grasper position (0 is retracted, 1 is protracted)
x_{sw}	0	seaweed position (positive is away from the animal)

Table 4. *Aplysia* feeding model initial conditions, reproduced from (Wang et al., 2022).

REFERENCES

Butera, R. J., Rinzel, J., and Smith, J. C. (1999). Models of respiratory rhythm generation in the pre-Bötzinger complex. I. Bursting pacemaker neurons. *Journal of Neurophysiology* 82, 382–397. doi:10.1152/jn.1999.82.1.382. PMID: 10400966

Diekman, C. O., Thomas, P. J., and Wilson, C. G. (2017). Eupnea, tachypnea, and autoresuscitation in a closed-loop respiratory control model. *Journal of Neurophysiology* 118, 2194–2215. doi:10.1152/jn.00170.2017

Ermentrout, G. B. and Terman, D. H. (2010). The Hodgkin–Huxley equations. In *Mathematical Foundations of Neuroscience* (Springer). 1–28

Hines, M. L. and Carnevale, N. T. (2004). Discrete event simulation in the NEURON environment. *Neurocomputing* 58, 1117–1122

Hlastala, M. P. and Berger, A. J. (2001). *Physiology of Respiration* (Oxford University Press)

Keener, J. and Sneyd, J. (2009). *Mathematical Physiology: II: Systems Physiology* (Springer)

Kim, H. (2017). Muscle length-dependent contribution of motoneuron Cav1. 3 channels to force production in model slow motor unit. *Journal of Applied Physiology* 123, 88–105

Kim, H. (2020). Linking motoneuron pic location to motor function in closed-loop motor unit system including afferent feedback: a computational investigation. *Eneuro* 7

Kim, H. and Heckman, C. (accepted). A dynamic calcium-force relationship model for sag behavior in fast skeletal muscle plos. *PLoS Computational Biology*

Lyttle, D. N., Gill, J. P., Shaw, K. M., Thomas, P. J., and Chiel, H. J. (2017). Robustness, flexibility, and sensitivity in a multifunctional motor control model. *Biological Cybernetics* 111, 25–47

Shaw, K. M., Lyttle, D. N., Gill, J. P., Cullins, M. J., McManus, J. M., Lu, H., et al. (2015). The significance of dynamical architecture for adaptive responses to mechanical loads during rhythmic behavior. *Journal of Computational Neuroscience* 38, 25–51

Wang, Y., Gill, J. P., Chiel, H. J., and Thomas, P. J. (2022). Variational and phase response analysis for limit cycles with hard boundaries, with applications to neuromechanical control problems. *Biological Cybernetics* 116, 687–710. doi:10.1007/s00422-022-00951-8

West, J. B. (2008). *Pulmonary Pathophysiology: The Essentials* (Lippincott Williams & Wilkins)

Yu, Z. and Thomas, P. J. (2021). Dynamical consequences of sensory feedback in a half-center oscillator coupled to a simple motor system. *Biological Cybernetics* 115, 135–160. doi:10.1007/s00422-021-00864-y
